# In silico identification of novel PTP1B inhibitors by the investigation of molecular mechanism, QSAR, and DFT studies

**DOI:** 10.1007/s40203-026-00649-w

**Published:** 2026-07-22

**Authors:** Humaira Zulfiqar, Samira Akter, Jarin Tasnim, Muhammad Younis, Md. Mehedi Hasan, Sidra Manzoor, Sarnop Sarker, Muhammad Waqas, Khan Rajib Hossain

**Affiliations:** 1 Department of Chemistry, COMSATS University Islamabad, Abbottabad, 22044 Pakistan; 2 Department of Pharmacy, East West University, Dhaka, 1212 Bangladesh; 3 Department of Applied Chemistry and Chemical Engineering, University of Rajshahi, Rajshahi, 6205 Bangladesh; 4 Department of Physics, Gomal University, Dera Ismail Khan, 29220 Pakistan; 5 Department of Chemistry, University of Agriculture, Faisalabad, 38000 Pakistan; 6 Department of Natural Science, BGMEA University of Fashion and Technology, Dhaka, 1230 Bangladesh; 7 Materials Engineering, University of Chinese Academy of Sciences, Beijing, 100040 China

**Keywords:** PTP1B inhibitors, Type 2 diabetes mellitus, Virtual screening, ADMET, Docking, DFT

## Abstract

Protein tyrosine phosphatase 1B (PTP1B) is a well-known and promising drug target involved in the negative regulation of insulin and leptin signaling, and new anti-diabetic molecules for the treatment of type 2 diabetes are directly in its hands. A series of PTP1B inhibitors was discovered and characterized using in silico techniques, including virtual screening, molecular docking, ADMET profiling, toxicity prediction, VEGA-QSAR analysis, molecular dynamics (MD) simulation, and density functional theory (DFT) calculations. A starting library of 1000 anti-diabetic compounds was screened, and PubChem CID 44560696 was identified as the most successful hit. The most potent inhibitor was then identified as CID 44560744 through chemical analogy-based refinement, with a binding free energy of − 9.13 kcal/mol and the ability to form several stabilizing hydrogen bonds and hydrophobic interactions with the key catalytic residues. DFT-optimized geometry and docking revealed that the lead compound shows high stability and strong PTP1B inhibition, showing superior binding affinity compared to ursolic acid, which exhibited a lower binding energy of − 6.34 kcal mol^−1^. The MD simulations showed that the PTP1B-ligand complex was structurally stable and compacted during the 200 ns simulation, with desirable profiles for RMSD, RMSF, Rg, SASA, and hydrogen bonds. The DFT and FMO analyses indicated that the compound exhibits extreme chemical reactivity and electron-transfer capability, owing to its low HOMO-LUMO gap, high softness, and moderate electrophilicity. The results of ADMET and toxicity evaluations (SwissADME, ProTox-II, and VEGA-QSAR) pointed to satisfactory solubility, non-carcinogenicity, no mutagenicity, and high pharmacological safety. Overall, the synergy of the computational methods has made CID 44560744 a lead scaffold for the development and optimization of selective PTP1B inhibitors for T2DM therapy.

## Introduction

**Fig. 1:**
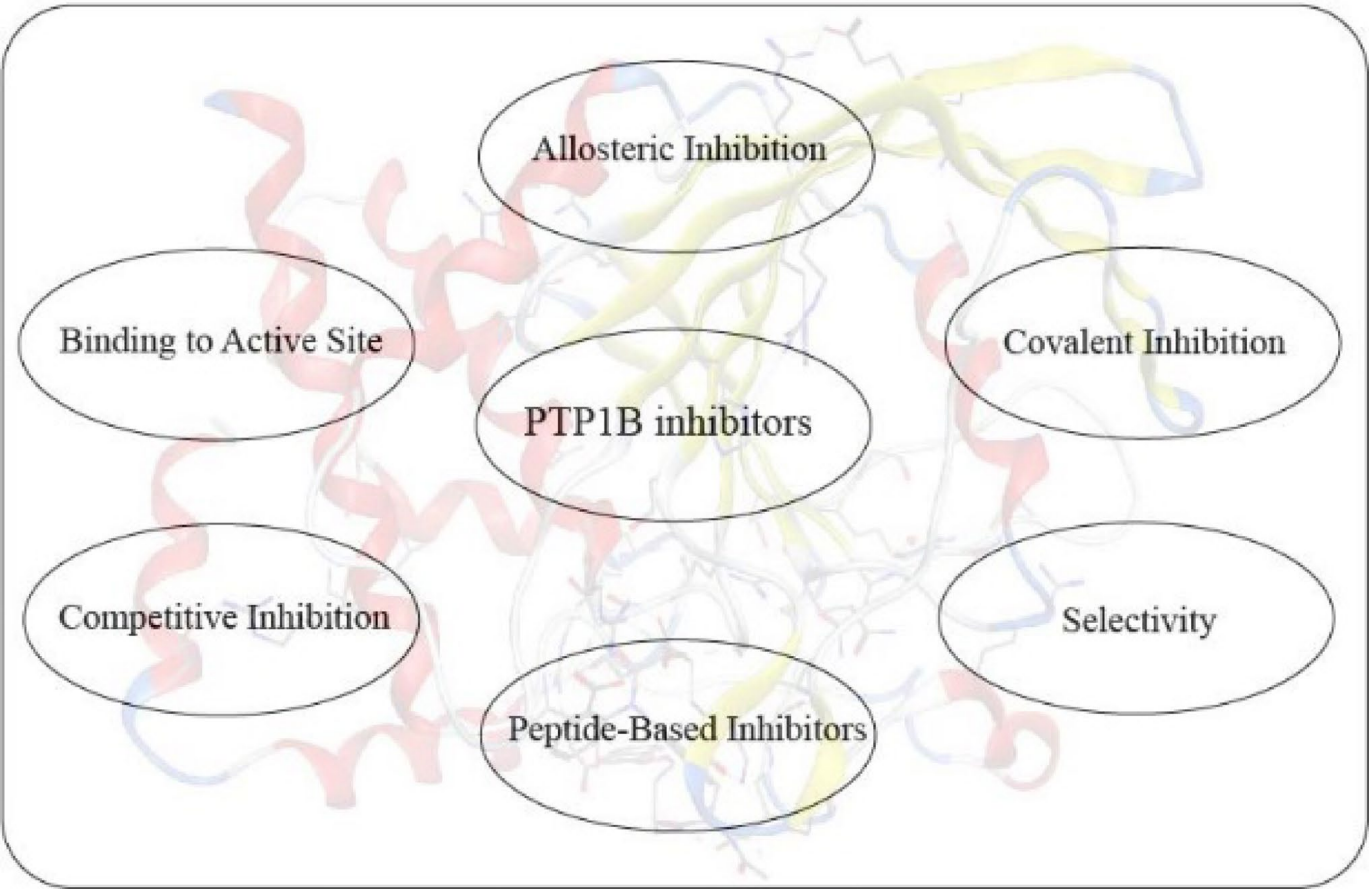
This diagram summarizes the various inhibitory mechanisms targeting PTP1B, including active-site binding, competitive inhibition, allosteric inhibition, covalent inhibition, peptide inhibition, and strategies to improve selectivity. These mechanisms constitute the main research directions for developing highly efficient and selective PTP1B inhibitors

Type 2 Diabetes Mellitus (T2DM) is the most common heterogeneous metabolic disorder, characterized by chronic hyperglycemia and disturbances in carbohydrate, lipid, and protein metabolism (Galicia-Garcia et al. 2020; Kumar et al. 2020). The major contributing factors include enhanced hepatic glucose synthesis, impaired insulin secretion, and reduced insulin sensitivity in peripheral tissues (Zhao et al. 2023; Lee et al. 2022). Numerous enzymes are involved in T2DM pathophysiology at the molecular level, such as protein tyrosine phosphatase 1B (PTP1B), DPP-4, α-glucosidase, and α-amylase (Proença et al. 2022). PTP1B, one of which has become a particularly favored drug target based on its pivotal role in dampening insulin and leptin signaling. PTP1B is a crucial protein tyrosine phosphatase regulating the insulin and leptin signaling pathways and is considered a potential therapeutic target for metabolic diseases and certain cancers. Figure 1 outlines the main design strategies for PTP1B inhibitors, including allosteric inhibition, active-site binding, competitive inhibition, covalent inhibition, selective optimization, and peptide inhibitors (Villamar-Cruz et al. 2021; Kumar et al. 2020). PTP1B inhibition improves insulin sensitivity and glucose metabolism, offering a promising therapeutic strategy for diabetes and obesity (Coronell-Tovar et al. 2024). The PTP1B inhibitory mode is through the inhibition of the enzyme activity to block the deep catalytic pocket (~ 8–9 Å) or sub-pockets contiguous to this pocket (Devi et al. 2024). These strategies comprise binding to the active site, competitive inhibition, allosteric modulation, covalent inhibitors, and peptide-based methods (Tiemann and Rademann 2023; McCullough 2020). The A, B, C, and D pockets serve as the binding sectors of chemical compounds, which are involved in the activity (active site-A site), which includes essential residues Asp181, Gln262, Tyr46, Arg221, and Cys215, which contribute also B, C, D sites for selectivity or potency (protagonist), respectively (Liu et al. 2022; Chen et al. 2021).

**Fig. 2:**
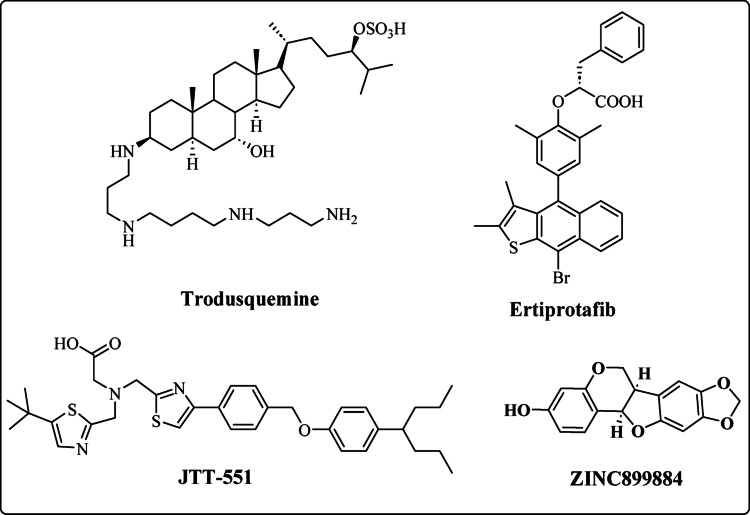
Chemical structures of typical PTP1B inhibitors that have entered clinical trials. This figure shows three typical PTP1B inhibitors: Trodusquemine (with allosteric inhibitory properties), Ertipurotafib (which has entered Phase II clinical trials), and JTT-551 (a synthetic inhibitor with insulin-sensitizing effects). ZINC899884 with the top binding energy value − 8.5 kcal mol^−1^. These representative skeletons provide an essential structural basis for the design and optimization of subsequent PTP1B-targeted drugs

Amino acid residues such as Gln262 and Asp181, along with hydrophobic residues (Tyr46, Arg221, Cys215, Ala217, Phe182, Ile219, Val49), determine enzymatic activity and inhibitor potency (Kabra and Khona 2024). The B site, a non-catalytic pocket, includes residues Arg254, Arg24, Ala27, Tyr20, Met258-Gly259, and Phe52 (Derki et al. 2024). The C site, a small and flat charged surface connected to the A site by Tyr46 and Asp48, disrupts the π-stacking of Trp44 and destabilizes it in the absence of an inhibitor, thereby improving inhibitor selectivity through salt bridges and electrostatic interactions (Ercan et al. 2025). The D site also shares polar amino acids (Glu115, Asp181, Ser216, Tyr46, and Lys120) and is involved in potency, but it does not bind insulin receptor kinase peptides directly (Zhang et al. 2023). Despite extensive studies over the past two decades, identifying PTP1B inhibitors remains challenging due to a well-conserved catalytic site shared with other protein tyrosine phosphatases (e.g., TCPTP shows ~ 80% identity) (Perez-Quintero et al. 2024; Klebe 2025). Only a few compounds, such as trodusquemine, ertiprotafib, and JTT-551, have been involved in clinical testing (Fig. 2). Ertiprotafib was studied in phase 2, but its development was discontinued due to adverse effects and lack of efficacy (Delibegović et al. 2024). A 2024 virtual screening of ZINC natural compounds led to the discovery of multiple PTP1B binders with optimal drug-likeness, thus affirming natural scaffolds as lead structures (e.g., ZINC899884) with low binding energy − 8.5 kcal mol^−1^. Trodusquemine and JTT-551 were effective in phase 1 studies, but did not progress to phase 2 research on subjects with T2DM because of side effects. A 2024 virtual screening of ZINC natural compounds found several PTP1B binders with favorable drug-like properties, reinforcing natural scaffolds as lead compounds (e.g., ZINC899884) with binding energies of − 8.5 kcal mol^−1^ (Fig. 2) (Singh and Thareja 2024)

The major bottleneck in the discovery of PTP1B inhibitors is balancing PTP1B selectivity, cellular permeability, and oral bioavailability. The existence of structural homology with other phosphatases makes drug design difficult, although the PTP sub-pockets near the active site can be exploited to increase selectivity (Thornton 2022). Developments in computational biology and CADD have greatly revolutionized contemporary drug discovery, enabling rapid, low-cost discovery of promising therapeutic agents (Tiwari and Singh 2022; Askari et al. 2023). Of these methods, molecular docking, along with virtual screening, is widely used to predict binding affinity and interaction patterns between bioactive compounds and a particular protein target (Mohammad et al. 2020; Zhang et al. 2024). Integration of these tools, together with ADMET prediction, toxicity profiling, VEGA QSAR, and density functional theory (DFT) calculations, provides a robust platform for screening drug candidates before experimental validation (Hussein and Azeez 2023; Banerjee et al. 2024).

Herein, we aimed to discover a new inhibitor of protein tyrosine phosphatase 1B (PTP1B). The library of anti-diabetic compounds was initially selected via virtual screening. Docking studies were subsequently conducted to investigate intermolecular interactions between these ligands and the active sites of PTP1B, focusing on compounds with good docking scores and binding energies, as well as stable hydrogen bonding or hydrophobic interactions. The top-ranked docked compound was further screened using ADMET and drug-likeness criteria, per Lipinski’s rule of five, to predict its pharmacokinetic properties and probable safety profile. Further, to confirm and optimize the results from the strategies used, molecular dynamics simulations of the top hit molecules were performed to evaluate the conformational stability of the protein–ligand complexes. In addition, frontier molecular orbital (FMO) and DFT-based studies have been conducted to investigate the electronic properties, reactivity descriptors, and global stability of these ligands. By combining these in silico methods, we hope to identify lead candidates against PTP1B with improved bioavailability, toxicity profiles, and stability, thereby shortening the discovery process for type 2 diabetes mellitus therapeutics.

## Materials and methods

### Protein and ligand preparation

Both the designed and reference compounds were docked into the active site of PTP1B (Akyol and Kilic 2021). The crystal structure of three-dimensional PTP1B (PDB ID: 1NNY; 2.40 Å resolution) was retrieved from the Protein Data Bank (Islam et al. 2025). The protein was structurally optimized with BIOVIA Discovery Studio, which added missing hydrogen atoms and residues, removed nonessential water molecules, and co-crystallized ligands, and then subjected the system to energy minimization to stabilize the structure (Shalayel et al. 2023). The final protein model was further used for molecular docking (Binmujlli 2024). Ligands with known or potential antidiabetic activity were obtained from the PubChem and ChEMBL databases in SDF format (Jayabal et al. 2023). The models were converted to PDB format and minimized to optimal geometry and correct conformation for the following docking analysis (Güner Yılmaz et al. 2025). 

### Virtual screening and molecular docking

Virtual screening is the most cost-effective and high-throughput in silico method for ligand discovery from large databases such as PubChem, ChEMBL, CHEBI, and ZINC (Liao et al. 2011). This approach reduces the number of compounds subjected to experimental testing, thus saving time and cost in drug discovery (Caldwell et al. 2001). Various techniques have also been utilized in virtual screening, such as ligand-based, structure-based, and hybrid methods, which all have their own advantages for identifying potential lead compounds (Devi et al. 2024). Notably, reverse-sequential screening with virtual structure-based docking followed by similarity-based analogue searching has been successfully used to optimize HTS hits into lead molecules (Szilágyi ). In this work, a structure-based virtual screening method was used to identify new inhibitors of protein tyrosine phosphatase 1B (PTP1B), an established target for type II diabetes mellitus. A set of about 1000 compounds was obtained from the PubChem and ChEMBL databases and docked into the binding site of PTP1B using AutoDock Vina via the PyRx interface. Binding energy and root mean square deviation (RMSD) values were calculated to check the affinity and stability of ligand-protein complexes. Subsequently, the highest-ranking compound in the initial screen was selected. To further optimize this screening and investigate structural analogs, the best inhibitor was used as a query template to search PubChem for similar compounds. Nine structurally similar compounds were prepared and docked under the same conditions. The inhibitor with the highest binding affinity among these analogues was selected as a likely stronger predicted binder than the lead compound. This two-step workflow for large-scale virtual screening and analogue-based optimization was developed to discover and rank potentially active chemical leads rapidly for further computational and experimental evaluation.

Molecular docking (protein-ligand) was conducted using PyRx (AutoDock Vina v0.8). The protein was constrained to be rigid during docking, while the ligands were considered flexible (Sankaranarayanan et al. 2025). The exhaustiveness parameter was fixed at 8, the grid box created at X = 30.781 Å, Y = 30.970 Å, and Z = 25.677Å, and energy minimization was conducted within the frame of the Universal Force Field (UFF) (Santos et al. 2023). Ligands and protein structures were converted into the PDBQT format through the Open Babel utility implemented in PyRx (Pavan and Shankar 2025). A box was created around the live binding site, with its dimensions and position personalized to cover the target pocket as entirely as possible (Ayodele et al. 2023; Kondapuram et al. 2021). The docking protocol used was a semi-flexible docking with a Broyden-Fletcher-Goldfarb-Shanno algorithm (Asim 2024). The docking protocol used AutoDock Vina, which employs a stochastic global search combined with local gradient optimization to predict ligand binding poses and binding affinities. AutoDock Vina returned multiple conformations, and the associated binding energies were saved in CSV format (Nguyen et al. 2019; Botková 2025). The docking results were further analyzed using Discovery Studio Visualizer (DSV 2024) to examine protein-ligand interactions (Behera et al. 2023). For each complex, the best conformer was selected based on docking score and favorable non-covalent interactions with key active site residues (Badrinarayan and Sastry 2014). 

### In silico MD-DFT-ADMET-Toxicity-VEGA-QSAR analysis

DFT calculations were performed using the DMol^3^ module in Materials Studio 2020 for the lead compound obtained from structure-based virtual screening. The DFT calculations were carried out to obtain the fully optimized geometry of the lead compound. Subsequently, molecular docking was re-performed using a DFT-optimized structure to achieve more reliable binding interactions, and an accurate binding conformation was obtained. DFT calculations performed using the DMol^3^ module in Materials Studio 2020. The hybrid with M06-2X was employed for Geometry optimizations and electronic structure calculations. The double numerical plus polarization (DNP) basis set (basis file 4.4) and fine numerical quality were used in the operation. SCF convergence was enforced for up to 300 iterations, with an energy tolerance of 1.0 × 10^−6^ and an electric smearing of 0.005 eV. HOMO and LUMO energies and their gap were taken from the converged single-point calculations. The total and projected density of states were also obtained from the converged wave functions using the same smearing parameters. Electrostatic potential (ESP) maps were generated by mapping the ESP onto the electron density iso-surface (Mohammadi et al. 2023). To gain insight into the compound’s chemical reactivity and stability, a Frontier Molecular Orbital (FMO) analysis was conducted. The energy of HOMO and LUMO was calculated, as well as the band gap between HOMO and LUMO, to evaluate the chemical stability of the inhibitor (Kumar et al. 2021). Furthermore, some global reactivity descriptors for IB were also computed, such as chemical potential (µ), chemical hardness (η), softness (S), and total electrophilicity index (ω) (Singh et al. 2023).

1 $$ \eta = (E_{{{\mathrm{LUMO}}}} - E_{{{\mathrm{HOMO}}}} )/2 $$

2 $$ \chi = - (E_{{{\mathrm{HOMO}}}} + E_{{{\mathrm{LUMO}}}} )/2 $$

3 $$ S = 1/(2\eta ) $$

4 $$ \mu = (E_{{{\mathrm{HOMO}}}} + E_{{{\mathrm{LUMO}}}} )/2 $$

5 $$ \omega = \mu ^{2} /(2\eta ) $$

These QCSA parameters are instrumental in understanding the stability, reactivity, and electrophilic nature of the proposed inhibitors (Latif et al. 2025). 

Molecular Dynamics Simulation (MDs) MDs of the binding affinity, structural flexibility, and entropic activity of protein-ligand and protein-standard complexes were analyzed using molecular dynamics simulation (MDs) under a synthetic environment (Sasidharan et al. 2023). However, before commencing the production simulations, energy minimization and equilibration simulations were performed to eliminate any unfavorable contacts between the solute and solvent molecules. During the first phase of energy minimization, harmonic restraints were applied to the solute and lipid chains, and in the second phase, all atoms were allowed to move freely (Grotz and Schwierz 2021). MD simulations were performed using GROMACS on Ubuntu, a Linux environment. Simulated moves were performed using the AMBER force field, one of the most widely supported and preferred choices for macromolecular systems (Xu et al. 2021). The PTP1B protein was used as the control to assess structural integrity against complexes. The periodic box was designed to ensure a 12 Å distance between the protein surface and the box boundaries, while short-range non-bonded interactions were calculated using a 15 Å cutoff. The simulation was performed at 300 K, 1 bar, and pH 7.0 (Tan et al. 2017). The steepest descent algorithm was used to minimize the system’s energy. Temperature and Pressure were maintained using a V-rescale thermostat and a Parrinello-Rahman piston, respectively (Pereira 2021). The simulation produced trajectories every 100 ps employing a 1.25 fs time step. Ligand topology was generated using the CHARMM force field 36, parameterized file obtained from swissparam. Several parallel trajectories were run for each complex to guarantee convergence. The duration of each MD simulation was 200 ns for the purpose of investigating the main factors, such as root mean square deviation (RMSD), root mean square fluctuation (RMSF), radius of gyration (Rg), solvent-accessible surface area (SASA), and the number of hydrogen bonds, hydrogen bond of ligand, RMSD of Ligand, PCA, and Covariance analysis. Matplotlib did the graphical representation of the resulting MD plots (da Silva et al. 2022). 

Pharmacokinetic (ADME) properties of the top-scoring docked compounds were calculated using the SwissADME web server (Daina et al. 2017). The SMILES representations of each compound were used to compute critical physicochemical descriptors, including drug-likeness, lipophilicity, solubility, and gastrointestinal absorption (Sucharitha et al. 2022). The toxicity profiles of the selected compounds were analyzed using the ProTox-II web server (Nnadi et al. 2020), which predicts multiple toxicological endpoints, including hepatotoxicity, mutagenicity, carcinogenicity, and cytotoxicity (Molla et al. 2023). The above-mentioned combined computational method yielded effective virtual screening of PK profiles, toxicities, and side effects, identifying safe and safety look-alike compounds for early-stage experimental validation (Pérez Santín et al. 2021). In silico photokinetic and toxicological parameters were estimated using the VEGA QSAR platform (Virtual models for property Evaluation of chemicals within a Global Architecture). It contains a set of QSAR models for calculating the physicochemical, pharmacological, and toxicological properties of small organic molecules (Gadaleta et al. 2024). In the present work, several vital endpoints, including skin irritancy potential, inhibitory activity against P-glycoprotein (P-gp), mutagenicity (Ames test), TBEH, and plasma protein binding, were assessed based on VEGA QSAR. The platform utilizes validated statistical and mechanistic modeling methodologies, in which molecular descriptors are fused with curated experimental datasets to produce predictive results, along with reliability indices (Banjare et al. 2021). Overall, the use of VEGA QSAR enabled a broad evaluation of ADMET and safety concerns supporting/corroborating computational profiling of the newly synthesized compounds (Nisa et al. 2023). 

## Results and discussion

### Virtual screening

**Fig. 3:**
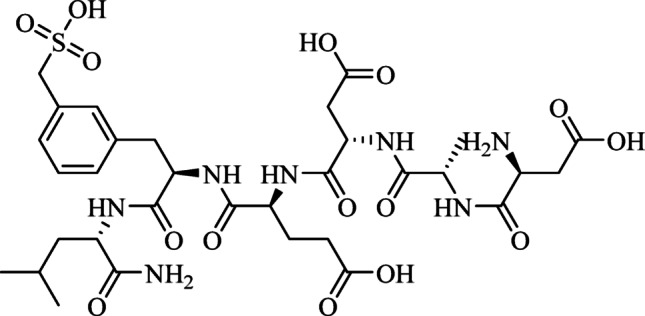
Structure of best lead compound (3 S,6 S,9 S,12 S,15R,18 S)-3-amino-18-carbamoyl-12-(2-carboxyethyl)-9-(carboxymethyl)-6,20-dimethyl-4,7,10,13,16-pentaoxo-15-(3-(sulfomethyl)benzyl)-5,8,11,14,17-pentaazahenicosanoic acid

The virtual screening was conducted against protein tyrosine phosphatase 1B (PTP1B), a known target for the treatment of type II diabetes mellitus. A library of 1000 compounds was first collected from the PubChem and ChEMBL databases, then submitted for docking with AutoDock Vina. The binding affinities were assessed to select promising lead candidates that effectively bind the catalytic site of PTP1B. PubChem CID 44560696 had the lowest binding energy of -8.3447 kcal/mol, indicating a stable, high-quality docking pose (RMSD: 1.7083 Å) among the selected compounds in this study. To further explore the structural potential of this hit compound, a structure-based analogue search was carried out in PubChem using CID 44,560,696((3 S,6 S,9 S,12 S,15R,18 S)-3-amino-18-carbamoyl-12-(2-carboxyethyl)-9-(carboxymethyl)-6,20-dimethyl-4,7,10,13,16-pentaoxo-15-(3-(sulfomethyl)benzyl)-5,8,11,14,17-pentaazahenicosanoic acid) as a query in Fig. 3. A total of nine structurally related analogues were retrieved and subjected to docking studies under identical conditions. Among these analogues, PubChem CID 44560744 (Fig. 1) demonstrated a significantly improved binding affinity of -9.1325 kcal/mol, with an RMSD of 2.0177 Å. This result indicates that CID 44560744 forms a more stable, energetically favorable complex with PTP1B than the initial lead (CID 44560696). The improvement in binding energy observed in CID 44,560,744 highlights the importance of scaffold modification and analogue screening in the identification of more potent inhibitors. The RMSD values obtained for both ligands (< 2.5 Å) are within acceptable limits, confirming the reliability of the docking poses. These findings suggest that CID 44560744 could serve as a promising candidate for further computational and experimental validation. Overall, the two-step approach, initially large-scale virtual screening followed by analogue-based refinement, proved effective in narrowing down potential PTP1B inhibitors. The identification of a novel lead-binding compound (CID 44560744) with high binding affinity confirmed its potential as a candidate for the development of new anti-diabetic drugs targeting PTP1B.

### DFT: frontier molecular orbitals (FMOs) and molecular electrostatic potential (MEP)

**Fig. 4:**
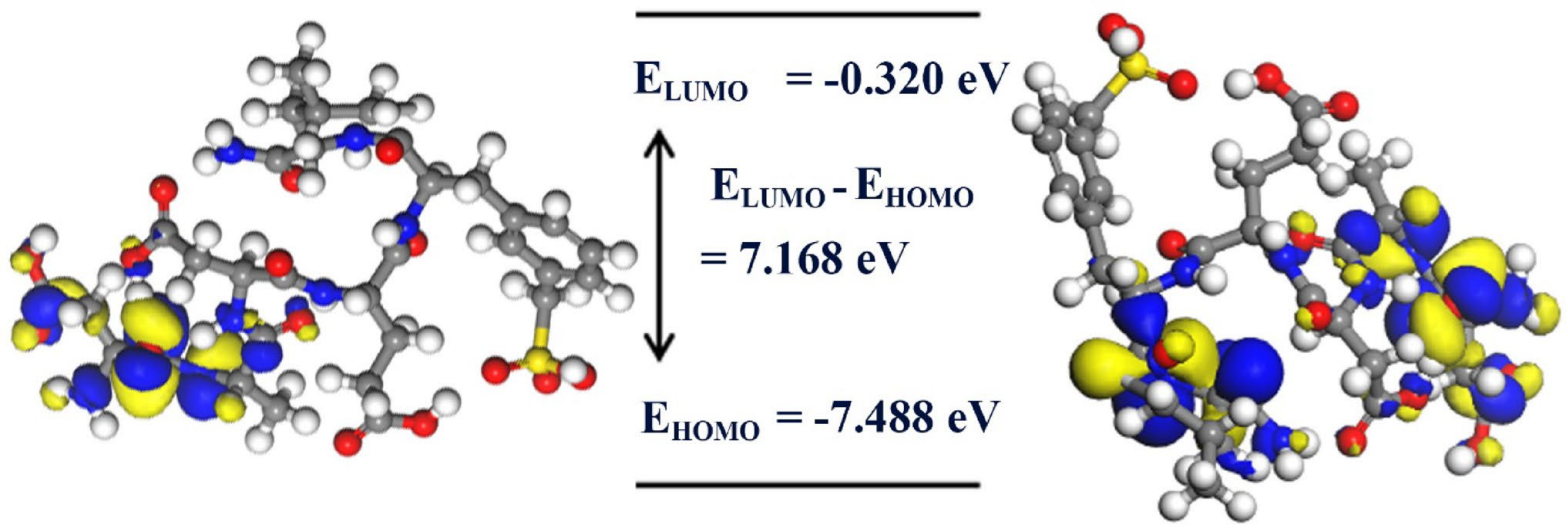
Distribution patterns of HOMO and LUMO orbitals of lead compounds

DFT calculations with the DMol^3^ module of Materials Studio 2020. Geometry optimization and electronic structure calculation. For geometry optimizations and electronic structure calculations, the hybrid with M06-2X was used (Chianelli et al. 2025). The electronic properties of the molecule, including Frontier Molecular Orbitals (FMOs), Electrostatic Potential (ESP), Density of States (DOS), and Partial Density of States (PDOS), have been studied. Global DFT descriptors were built based on the FMO energies, and the Lead Compound was found to possess high reactivity with lower structural stability. For reliable evaluation of GB structural features and geometrical optimization, we used DFT-based methods. The HOMO and LUMO orbital energies were employed to calculate the global chemical reactivity descriptors, such as the electronegativity (χ), chemical hardness (η), chemical potential (µ), softness (S), and electrophilicity index (ω). These parameters provide helpful information on the reactivity and stability of the compound shown in Table 1. The calculated E_LUMO_-E_HOMO_ was 7.168 eV, suggesting a very high band gap, consequently, high stability (Fig. 4).

**Table 1:** Lead compounds reactivity and Frontier orbitals energies values

Parameter	Symbol	Formula	Value (eV)
Chemical hardness	η	(E_LUMO_ − E_HOMO_)/2	3.584
Electronegativity	χ	− (E_HOMO_ + E_LUMO_)/2	3.904
Softness	S	1 / (2η)	0.139 eV^−1^
Chemical potential	µ	(E_HOMO_ + E_LUMO_)/2	− 3.904
Electrophilicity index	ω	µ² / (2η)	2.13

When a molecule has a low band gap, its electrons are not tightly bound and can therefore participate in electron-transfer reactions with less resistance. Thus, the molecule is characterized by high chemical reactivity and, to some extent, is kinetically stable. High band gap materials (over 3 eV), on the other hand, are generally considered to be unreactive and less likely to accept or donate electrons, whereas those with a medium band gap (0.5–3 eV) are assigned a range of reactivity from low to moderate. The computed ΔE for the compound is 7.168 eV, so it is labeled as a high band gap. Chemical hardness (η) was found to be 3.584 eV, and this number represents the resistance of the molecule toward charge transfer. A high η suggests that the molecule is soft and lower reactivity. The softness (S) of 0.139 eV^− 1^ means the reactivity of the compound is low, and less polarizable. The resultant values for the electronegativity (χ) and chemical potential (µ) are 3.904 eV and − 3.904 eV, respectively. If the chemical potential is negative, the system is in a thermodynamically stable state, though it may still act as an electron donor to other media. The electrophilicity index (ω) was calculated to be 2.13 eV, indicating that the molecule has electrophilic character. The medium ω value indicates a similar contribution to both the electrophilicity and nucleophilicity of the compound, which is evidence showing that the compound behaves as a moderate electrophile. The high band gap, moderate hardness, and moderate electrophilicity index also imply that compound could have controlled chemical reactivity and high stability.

**Fig. 5:**
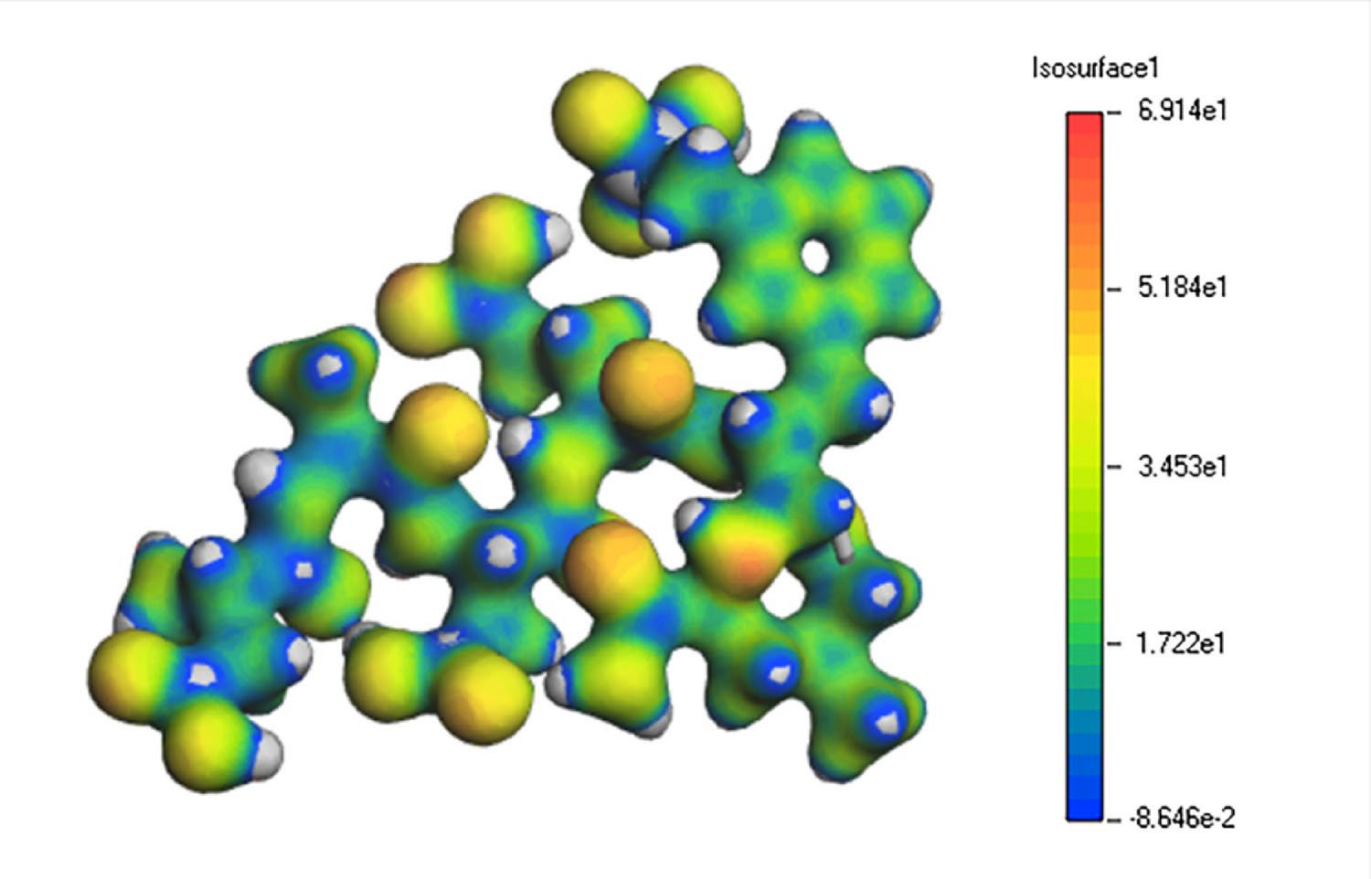
Illustrates the three-dimensional isosurface distribution of a molecular structure. Colored isosurfaces represent the spatial distribution of a scalar field (such as electron density or electrostatic potential) around the molecule, with values ranging from blue (low) to red (high) according to the color scale on the right. The ball-and-stick model shows the atomic arrangement and configuration of the molecule, providing a visual comparison of the relationship between structure and field distribution

The Molecular Electrostatic Potential (MEP) is used to predict reactive sites within a molecule for potential nucleophilic and electrophilic attack. In this study, the MEP surface of the compound was analyzed using Density Functional Theory (DFT) calculations, based on the optimized geometry. The resulting electrostatic potential map is illustrated in Fig. 5. The color scale of the MEP ranges from − 8.646e^−2^ to − 6.914e^1^, where red regions indicate electron-rich (nucleophilic) areas, and blue regions represent electron-deficient (electrophilic) zones.

#### Density of states (DOS), and partial density of states (PDOS)

**Fig. 6:**
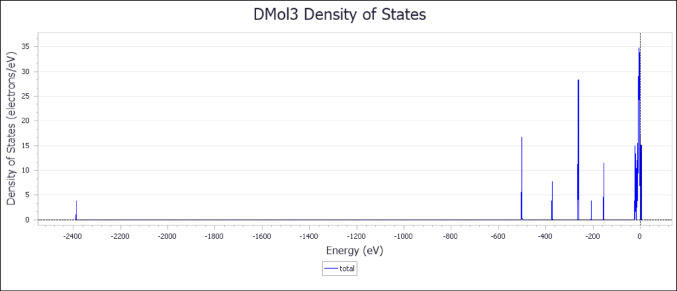
The DOS (density of states) obtained from DMol^3^ using the hybrid with M06-2X functional depicts the distribution of electronic states over the entire energy range. The peaks around the Fermi level (0 eV) reveal which molecular orbitals are active and can drive the compound’s electronic reactivity

The Density of States (DOS) graph generated by DMol^3^ presents the distribution of electronic states over a wide energy range. In addition, the graph shows several sharp peaks that denote highly localized electronic states, with the bulk of the significant features situated near the Fermi level (set to 0 eV) and in the higher-energy region. A high density of such peaks in this region indicates the active molecular orbitals involved in the compound’s electronic transitions and thus its reactive sites. Conversely, intense states (e.g., below − 500 eV) are shown as only slightly occupied and are associated with core-level orbitals that are totally uninvolved in chemical interactions. In other words, the DOS could be considered “a map” to better understand the electronic structure, showing which regions correspond to bonding, non-bonding, and anti-bonding states, and verifying molecular reactivity trends using the FMO analysis diagram **(**Fig. 6**)**.

**Fig. 7:**
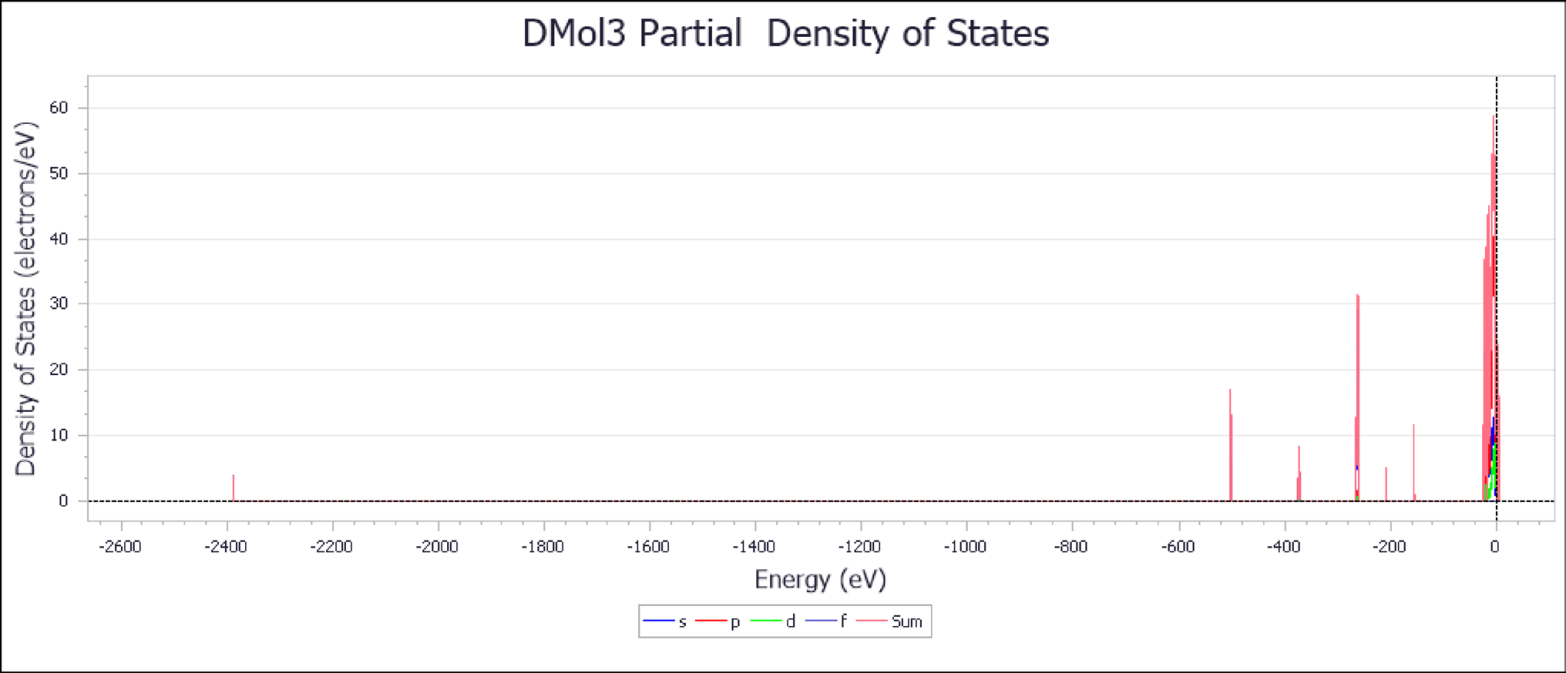
The PDOS (partial density of states) presents the contributions of s-, p-, d-, and f-orbitals towards the total DOS. The Fermi level area is marked by strong p-orbital contributions that emphasize their primary role in the molecule’s electronic interactions and chemical reactivity

The partial density of states (PDOS) was calculated using DMol3 (bulk option, Materials Studio 2020) with M06-2X to show the contribution of each orbital to the electronic structure of the molecule. PDOS is a technique that enables one to identify the atomic orbitals closest to the Fermi level and thus most influential in the molecule’s reactivity. According to the PDOS diagram, the p-orbitals have the greatest effect at the Fermi level (0 eV), indicating their leading role in electronic activity and chemical reactivity. The s- and d-orbitals contribute less, yet their contribution is still considerable. The peaks at deep energies are linked to core states that do not participate in bonding and are hence virtually non-reactive. Thus, it can be inferred that the interactions of the molecule’s p-orbitals are the chief factors that govern its reactivity (Fig. 7).

### Docking studies

**Fig. 8:**
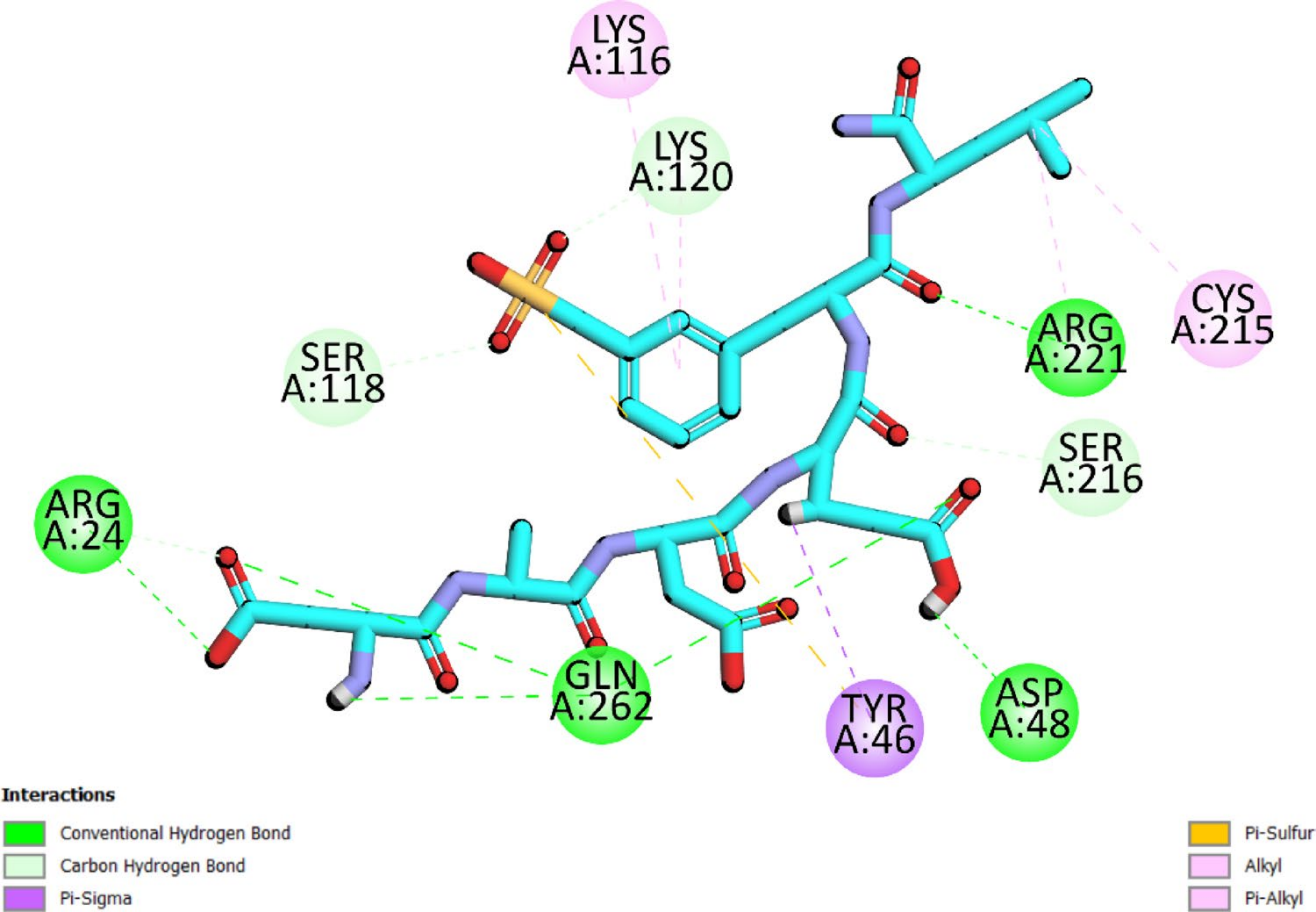
Shows the ligand’s binding modes to the target protein. Green dashed lines represent conventional hydrogen bonds and C–H hydrogen bonds, yellow represents π-sulfur interactions, and pink represents hydrophobic alkyl and π-alkyl, and purple represents π-sigma interactions. Amino acid residues involved in interactions (such as ARG24, ARG221, GLN262, ASP48, SER118, SER216, TYR46, CYS215, LYS116, and LYS120, etc.) are marked, indicating the ligand’s stable conformation at the binding site. (Color Code for Ligand: Blue-Carbon, Red-Oxygen, Dark Blue-Nitrogen, Yellow-Sulphur)

The docking research showed that the entity could be well situated in the catalytic pocket of PTP1B and thus bind to it with a calculated binding affinity of − 9.13 kcal mol^− 1^. Several stabilizing interactions were identified, including conventional hydrogen bonds with LYS116, ASP181, GLY183, ARG221, GLN262, and GLN266, which play an essential role in orienting the ligand at the binding site(Table 2). A hydrophobic alkyl association with LYS120 added to van der Waals stabilization. In contrast, a π-sulfur association with MET258 facilitated electronic complementarity, and these were associated with overall ligand binding stabilization. The complex was further stabilized by additional contacts to TYR20, ARG24, and ARG254 via hydrogen bonding and electrostatic interactions (Fig. 8). Of particular interest is that, as verified by the re-docking validation results, this protocol can be considered reliable because the AutoDock SP binding energy of the re-docked native ligand (− 10.0667 kcal mol^−1^) was similar to that of our testing molecule; thus, it was logically deduced that the predicted orientation pose and binding strength are credible. Taken together, these results demonstrate the excellent binding capacity of compound 11 for PTP1B.

**Table 2:** Theoretical molecular modeling of the interaction between the hit compound and active site residues of PTP1B

No.	Amino acid/group	Distance (Å)	Category	Type
1	ARG24	2.42	H-bond	Conventional H-bond
2	ARG24	2.14	H-bond	Conventional H-bond
3	LYS116	2.88	H-bond	Conventional H-bond
4	TRP179	2.39	H-bond	Conventional H-bond
5	GLY183	3.07	H-bond	Conventional H-bond
6	ARG221	2.19	H-bond	Conventional H-bond
7	ARG254	2.10	H-bond	Conventional H-bond
8	ARG254	2.63	H-bond	Conventional H-bond
9	GLN266	2.02	H-bond	Conventional H-bond
10	TYR20	2.72	H-bond	Conventional H-bond
11	GLN262	2.55	H-bond	Conventional H-bond
12	ASP181	2.08	H-bond	Conventional H-bond
13	ARG24	2.56	H-bond	Carbon H-bond
14	MET258	4.42	Other	Pi-sulfur
15	LYS120	4.46	Hydrophobic	Alkyl

**Fig. 9:**
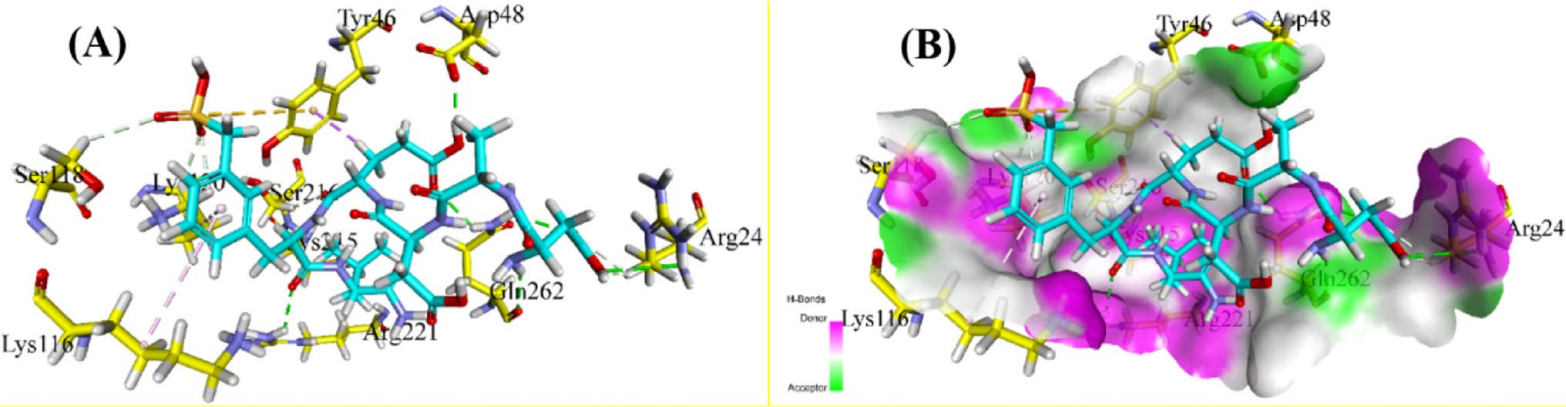
**A** Schematic diagram of the three-dimensional interaction distribution between the ligand and PTP1B (protein) binding site, emphasizing the surrounding support provided by amino acids such as ARG24, ARG221, GLN262, ASP48, and LYS120, etc.), and **B** Three-dimensional hydrogen bond donor and acceptor distribution at the ligand-target protein binding site. The molecular surface colors indicate hydrogen-bond interaction regions: pink represents hydrogen-bond donors, and green represents hydrogen-bond acceptors

Figure 9A shows the three-dimensional spatial arrangement of the ligand and PTP1B protein, emphasizing the surrounding support provided by amino acids such as ARG24, ARG221, GLN262, ASP48, SER118, SER216, TYR46, CYS215, LYS116, and LYS120. The molecular surface model in Fig. 9B further reveals the polar distribution of the binding cavity and the ligand’s adaptability; the pink and green areas represent hydrogen bond donors and acceptors, respectively, which help explain its stable binding mechanism. The re-docking verification shows that the AutoDock SP binding energy of the native ligand is − 10.1 kcal mol^−1^, which is close to the result for the target molecule, proving that the docking procedure in this study is reliable. Therefore, based on all the results, this compound shows excellent binding potential for PTP1B.

Lead compound exhibited an extended binding mode involving not only the conserved catalytic A pocket for catalytic inhibition, while simultaneously interacting with B, C, and D pocket residues. In A pocket, key contacts were established with GLN262, TYR46, CYS215, and ARG221, supporting effective catalytic site engagement. Additional interactions with ARG24 in the B pocket, TYR46, ASP48 in the C pocket, and SER216, TYR46, LYS120 in the D pocket highlight the compound’s ability to span multiple subsites. This multi-pocket binding profile suggests a selectivity-driven inhibition mechanism rather than inhibition confined solely to the catalytic site.

#### Docking studies of ursolic acid (standard compound)

**Fig. 10:**
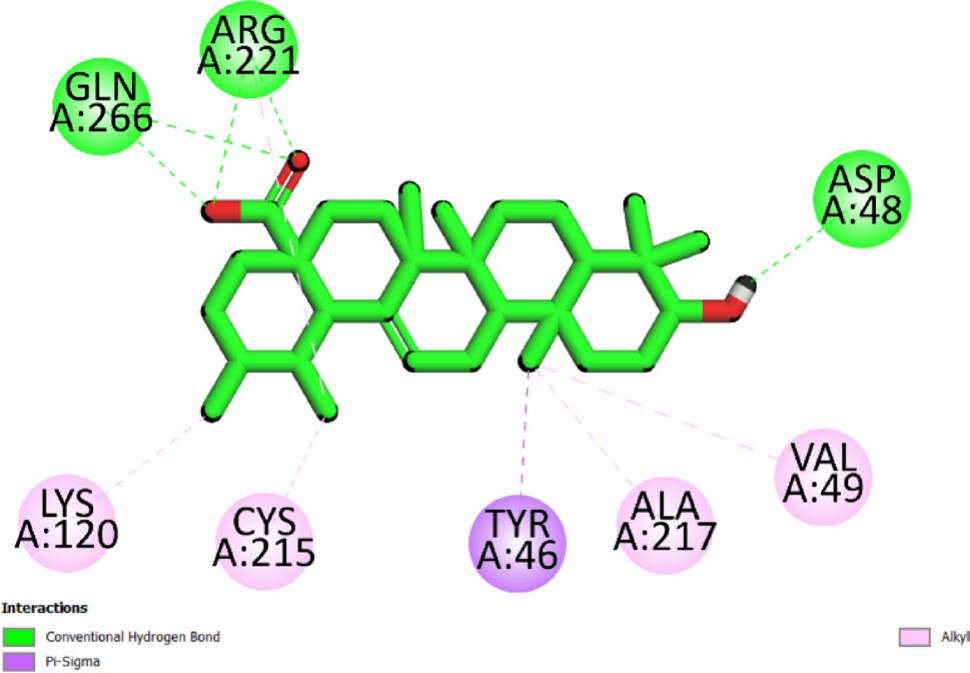
Shows the Ursolic acid binding modes to the target PTP1B (protein). Green dashed lines represent conventional hydrogen bonds and C–H hydrogen bonds, pink represents hydrophobic alkyl and purple represents π-sigma interactions. Amino acid residues involved in interactions (such as ARG, GLN, ASP, ALA, TYR, VAL, and CYS, etc.) are marked, indicating the ligand’s stable conformation at the binding site. (Color Code for Ligand:-Green-Carbon, Red-Oxygen)

**Fig. 11:**
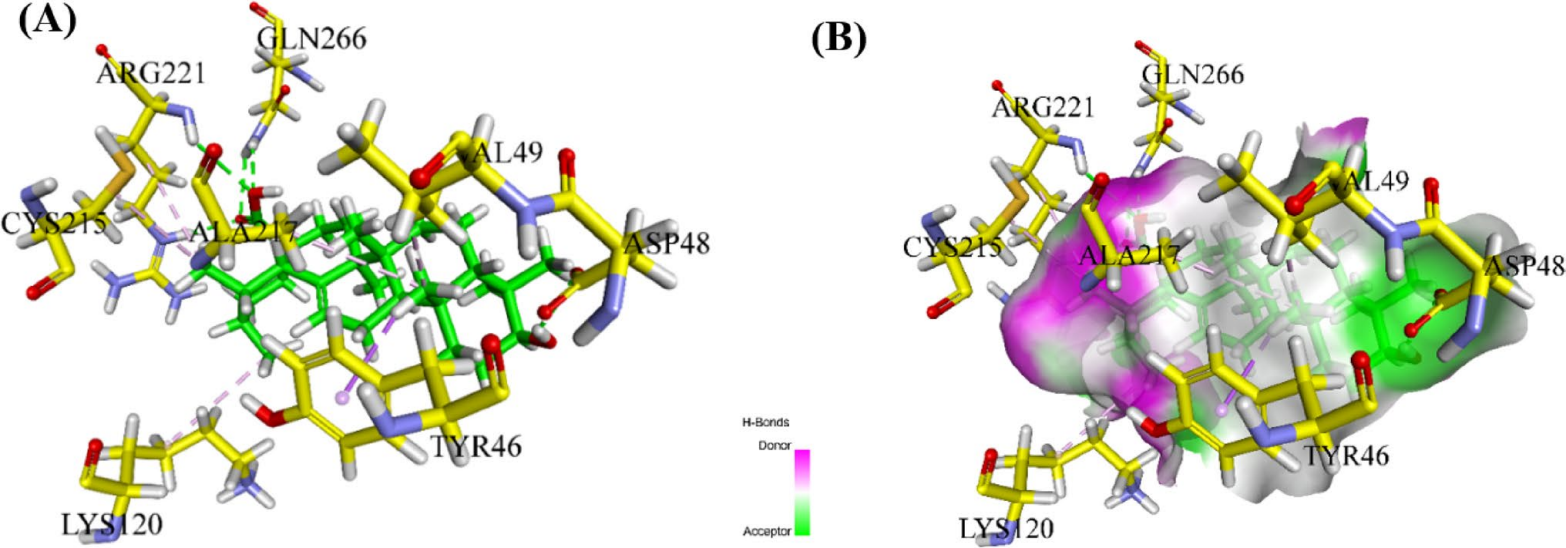
**A** Three-dimensional spatial arrangement of the Ursolic acid and PTP1B(protein), emphasizing the surrounding support provided by amino acids such as ARG221, GLN266, ASP48, TYR46, ALA217, VAL49 and CYS215, and **B** Three-dimensional hydrogen bond donor and acceptor distribution at the ligand-target protein binding site. The molecular surface colors indicate hydrogen-bond interaction regions: pink represents hydrogen-bond donors, and green represents hydrogen-bond acceptors

The docking research was also performed with standard inhibitor ursolic acid showed that the entity could be well situated in the catalytic pocket of PTP1B and thus bind to it with a calculated binding affinity of − 6.34 kcal mol^−1^. Several stabilizing interactions were identified, including conventional hydrogen bonds with ARG221, ARG221, GLN266, GLN266, and ASP48, which play an essential role in orienting the ligand at the binding site (Table 3). In contrast, a π-sigma association with TYR46 and ARG221 facilitated electronic complementarity, and these were associated with overall ligand binding stabilization. A hydrophobic alkyl association with ALA217, VAL49, CYS215 and ARG221 added to van der Waals stabilization (Figs. 10 and 11).

**Table 3:** Theoretical molecular modeling of the interaction between the reference compound and active site residues of PTP1B

No.	Amino acid/group	Distance (Å)	Category	Type
1	ARG221	2.73442	H-bond	Conventional H-bond
2	ARG221	2.05598	H-bond	Conventional H-bond
3	GLN266	2.86354	H-bond	Conventional H-bond
4	GLN266	2.29849	H-bond	Conventional H-bond
5	ASP48	2.15449	H-bond	Conventional H-bond
6	TYR46	2.94144	Hydrophobic	Pi–Sigma
7	ALA217	4.37503	Hydrophobic	Alkyl
8	VAL49	4.59220	Hydrophobic	Alkyl
9	CYS215	4.05239	Hydrophobic	Alkyl
10	ARG221	4.80356	Hydrophobic	Alkyl
11	ARG221	2.73442	Hydrophobic	Pi–Sigma

### Docking validation of native ligand with PTP1B

**Fig. 12:**
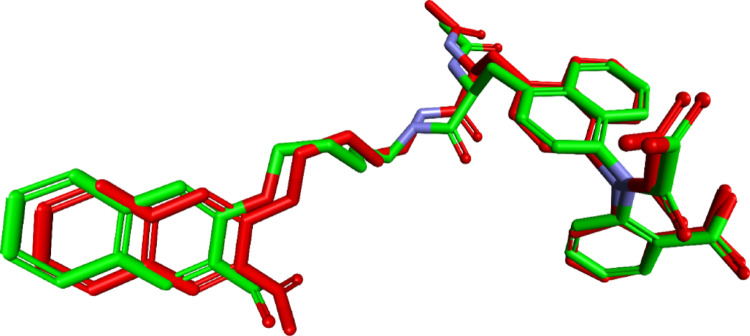
Overlay of the crystallographic ligand (red) and re-docked ligand (yellow) into the active site of PTP1B. Significantly, the extensive overlap between the two conformations reproduces the experimentally determined binding pose, supporting the validity and robustness of the docking protocol

**Fig. 13:**
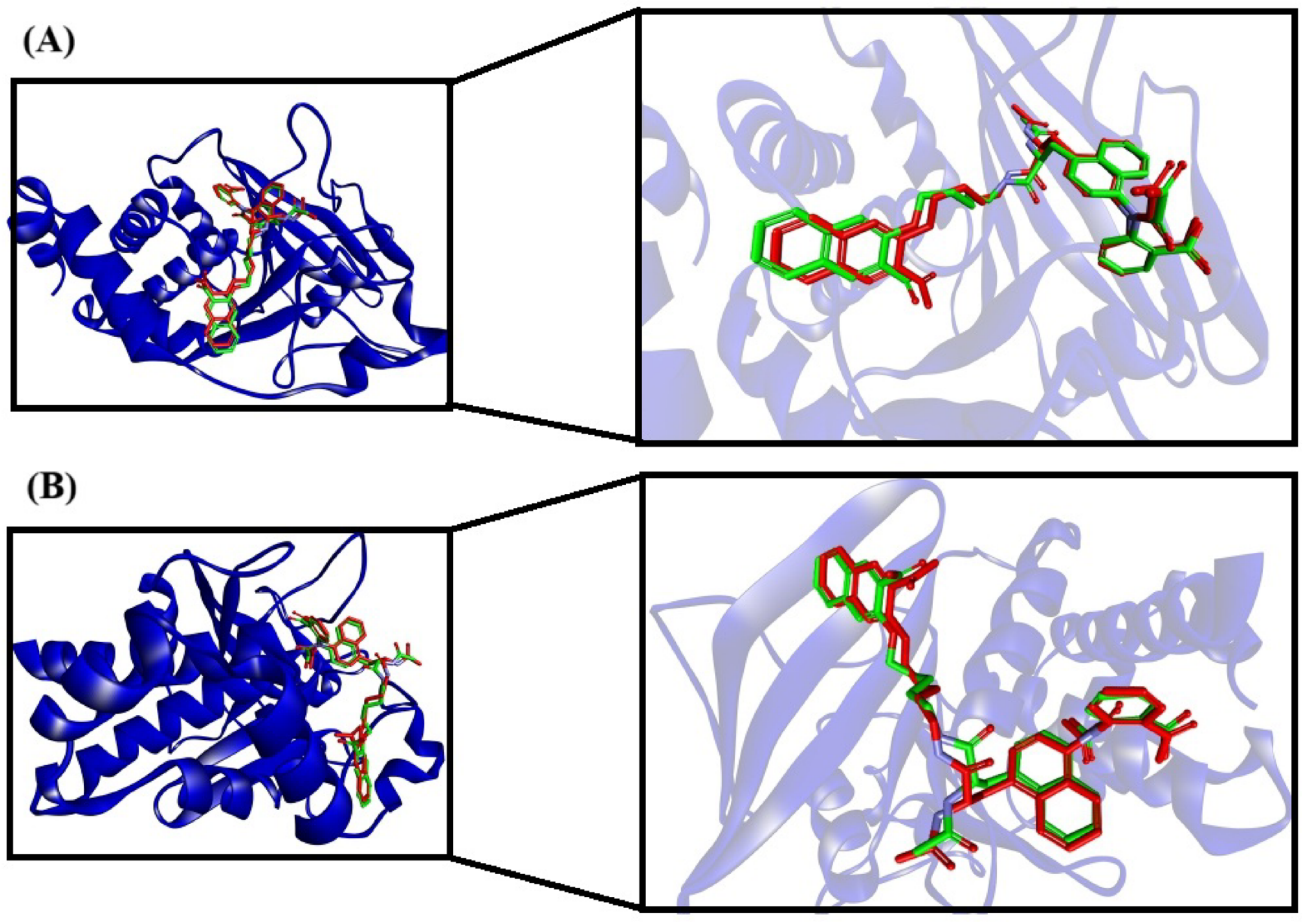
Validation of the docking protocol by re-docking the native ligand into the active site of PTP1B (PDB: 1NNY). The enzyme backbone is shown as a cartoon (green), with the docked ligand (yellow) superposed on the crystallographic ligand (red). **A** Side-view orientation of the active site and the close alignment between the docked conformation and the crystal structure. **B** Opposite side view of the same complex with ligand in its binding site. The close structural similarity of the two conformations is evidence for the robustness and transferability of the docking

The native ligand (co-crystallized in the active site) in PTP1B (PDB ID: 1NNY) was re-docked to confirm the docking methodology. The docked pose showed a high degree of overlap with its known crystallographic orientation; therefore, the docking methodology was validated. The good overlap between the re-docked and crystallographic conformations shows that we were able to properly optimize the docking procedure and predict ligand binding with high reliability at the same active site (Fig. 12). Such validation is mandatory for the estimation of the docking protocol reliability and stability that will be adopted in further binding affinity and interaction analyses, as shown in Fig. 13.

### MD simulation

**Fig. 14:**
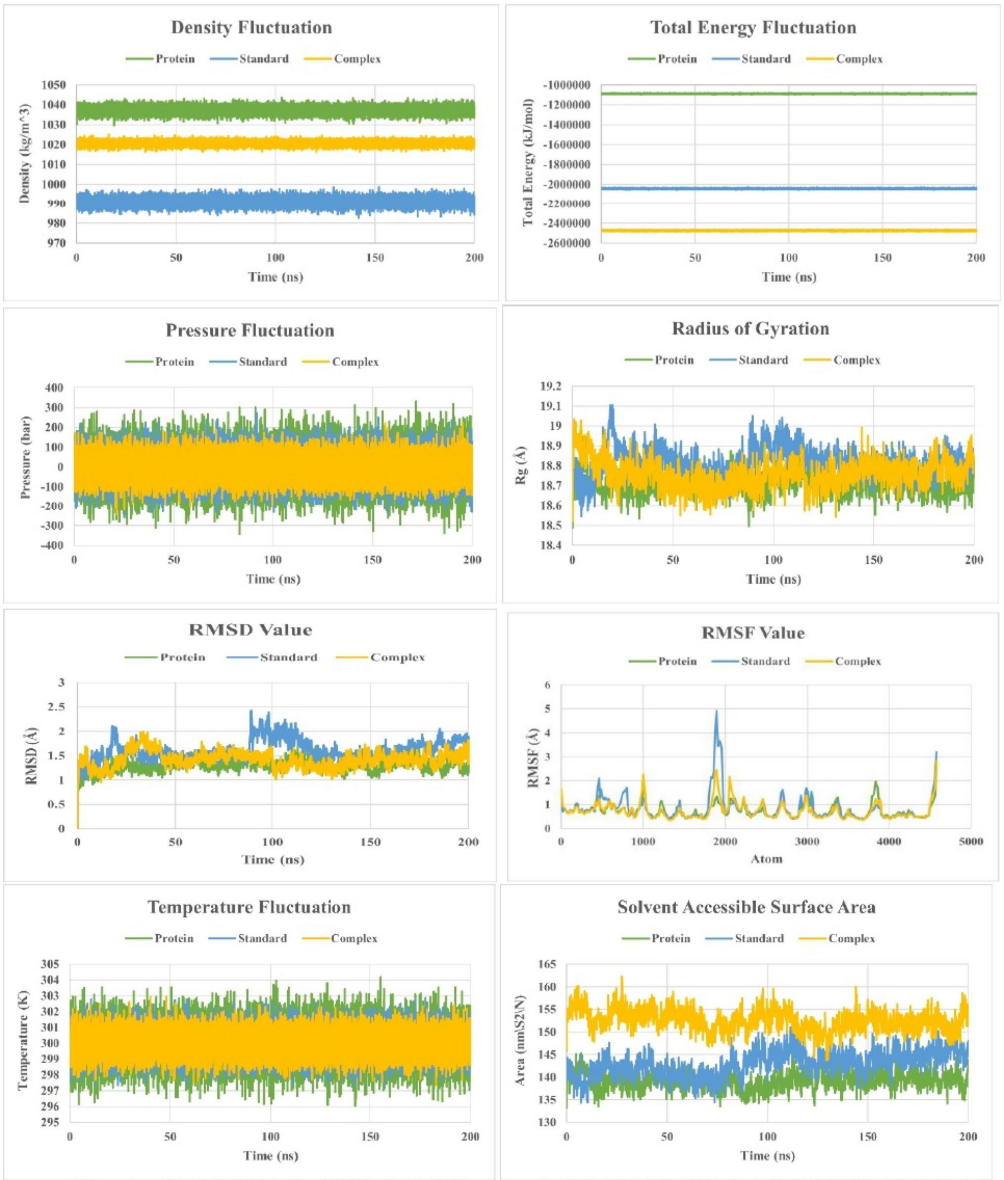
The lead and standard compound complex was subjected to RMSD, RMSF, Rg, and SASA analysis. Each MD simulation was executed for 200 ns for all systems. The corresponding MD trajectories were assessed through the data obtained from RMSD, RMSF, Rg, and SASA. The alpha carbons RMSD, residues flexibility RMSF, rigidity, and compactness Rg, as well as the protein volume with expansion SASA analyses, were used

MD simulation is an essential post-docking tool for elucidating the temporal stability and atomic movements of the lead compound, and standard compound within the active site of PTP1B. In the present work, we carried out two MD simulations on the apo protein and the PTP1B-ligand complex, and the PTP1B-reference complex, which were downloaded. From simulations, we gained insights into the structural dynamics, binding mechanism, and flexibility of both the apo protein and the complex. The analysed parameters are the following: RMSD, RMSF, Rg, SASA, and hydrogen bonding after a 200 ns run (Fig. 14). The latter kind of stability measurements is crucial for probing the strength of binding and the conformational integrity of the produced complexes.

#### RMSD Analysis

RMSD analysis for the apo protein PTP1B indicated slight variation between 45 ns and 67 ns, before equilibrating. There was another slightly higher RMSD peak for the complex’s RMSD, but it was just for a few nanoseconds, around 35–45 ns duration. This behaviour is very common in biomolecular MD simulations. It may reflect to transient regions of the protein or the ligand, but after 45ns, the RMSD became stable again. With respect to the PTP1B-ligand complex, a conformational change was also noted between 8 ns and 35 ns, following an initial stabilisation, reflecting integrity at the end of the simulation. The PTP1B-standard complex exhibited a conformational change was also noted between 2 ns and 38 ns, RMSD pattern throughout simulation, with minor fluctuations comparable to those of the lead-complex. The RMSD values remained within an acceptable range, suggesting that the ligand compound and standard compound formed a stable complex with PTP1B and did not induce significant structural destabilization. A comparison with the apo PTP1B protein indicated that, at least for the conformations sampled during simulation, the complex was stable.

#### RMSF analysis

RMSF analysis showed similar motion behavior for the apo protein with its native ligand. RMSD values of the receptor with the reference ligand were higher slightly than the apo protein and with the ligand in this study. This indicates that the ligand in the study is actually more stable than the reference ligand as well. As RMSF values for receptor with the reference ligand were approximately higher upto 2 angstrom but the ligand under study introduced less changes in it as the receptor’s RMSF values were lower than 1 angstrom. The lead compound demonstrated RMSF behavior moderately matching that of the standard compound, suggesting comparable dynamic restraint within the catalytic pocket.

#### SASA analysis

The average SASA of the apo protein, around 142 nm^2^, suggested that it remained in a stable, compact state during the simulation. In contrast, the complex also showed no considerable higher peaks in the SASA trajectory, indicating transient conformational extension during the simulation’s initial stage. It presented a mean SASA of ~ 155 nm^2^, indicating surface reshaping on ligand binding. In general, the steady stabilization of the SASA of the ligand-complex after 25 ns suggests that the overall structural compactness decreases over the simulation time. The steady stabilization of the SASA of reference-complex suggests that the overall structural compactness decreases over the simulation time. After 50 ns, SASA values for both lead and reference complexes stabilized, indicating formation of stable protein-ligand assemblies.

#### Radius of gyration (Rg)

To predict overall compactness and structural stability, we calculated the radius of gyration (Rg) for both the apoprotein and the PTP1B-ligand complex, and the PTP1B-reference complex. The value of Rg for the apo protein was found to be larger than that of the complex and reference. The lead complex maintained Rg values comparable to or slightly lower than the reference complex, highlighting its favorable stabilizing effect on protein structure. The lower Rg value of the complex, as compared to that of the apo protein, and its continued decrease over the trajectory, reveal the compactness and enhanced stability of the bound state. Considering the results of all the calculations, it seems clear that, despite the relatively small conformational rearrangements observed for PTP1B across all our simulations, the complex between the protein and one of its natural ligands is relatively rigid. The superimposed structures pre- and post-simulation with the simulated ligand confirm a conformational difference in the binding cavity before and after the simulation. Furthermore, the surface representation of the complex was generated at 25, 50, 75, and 100 ns, revealing gradual structural changes throughout the trajectory. In conclusion, the collective findings suggest that the PTP1B-ligand complex maintained structural stability throughout the simulation.

#### Hydrogen bond (H-bond)

**Fig. 15:**
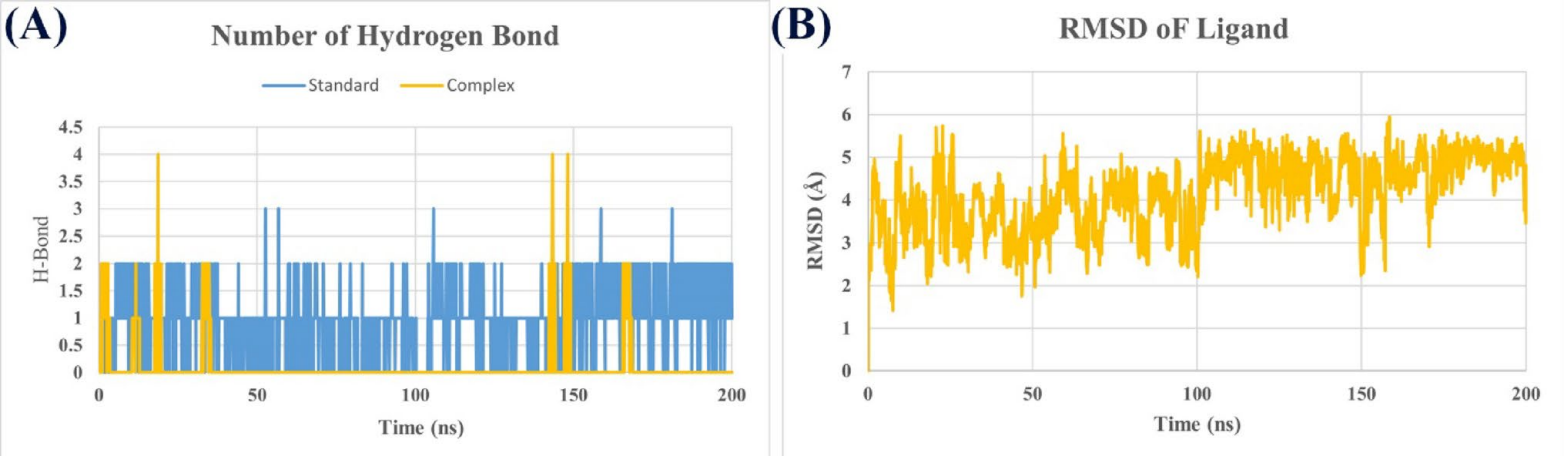
**A** The hydrogen bond of the ligand as a function of simulation time, showing persistent intermolecular interactions throughout the trajectory, and **B** RMSD of ligand relative to initial equilibrated structure, indicates early conformational relaxation followed by the stable fluctuations without long-term drift, consistent with the structurally stable bound conformation

The hydrogen bond (H-bond) profile of the ligand in explicit solvent was monitored over a 200 ns molecular dynamics simulation to evaluate its solvation behavior and physicochemical stability. The ligand consistently formed approximately 1–2 hydrogen bonds with surrounding solvent molecules throughout the simulation, with moderate fluctuations around a stable mean value (Fig. 15A). While some peaks went up to 4 hydrogen bonds as well from time to time for the complex. The persistence of solvent-mediated hydrogen bonding indicates that the ligand maintains a favorable balance between polar and nonpolar functional groups, enabling effective interaction with the aqueous environment. Importantly, no prolonged reduction or loss of hydrogen bonding was observed, suggesting the absence of aggregation or precipitation tendencies. The observed fluctuations are characteristic of a dynamically solvated small molecule and reflect natural rearrangements of hydrogen bond donors and acceptors rather than structural instability. Overall, the stable hydrogen bonding pattern supports good aqueous solubility and physicochemical robustness, both of which are critical attributes of drug-like compounds and are often associated with improved bioavailability.

#### RMSD of ligand

The structural stability of the ligand during the simulation was assessed by calculating its root mean square deviation (RMSD) relative to the initial equilibrated structure. As depicted in Fig. 15, the ligand RMSD increased rapidly during the initial phase of the simulation, corresponding to conformational relaxation from the starting geometry, and subsequently stabilized within a range of approximately 4.5–5 Å for the remainder of the 200 ns trajectory (Fig. 15B). The absence of a continuous upward drift in RMSD indicates that the ligand does not undergo progressive conformational destabilization or unfolding over time. Instead, the ligand samples a stable conformational ensemble, reflecting an optimal balance between structural rigidity and flexibility. Such behavior is desirable for small-molecule therapeutics, as conformational adaptability facilitates effective accommodation within protein binding sites while maintaining molecular integrity. Collectively, the RMSD profile suggests that the ligand remains structurally stable under physiological simulation conditions, supporting its suitability as a drug-like molecule.

#### Principal component analysis (PCA)

**Fig. 16:**
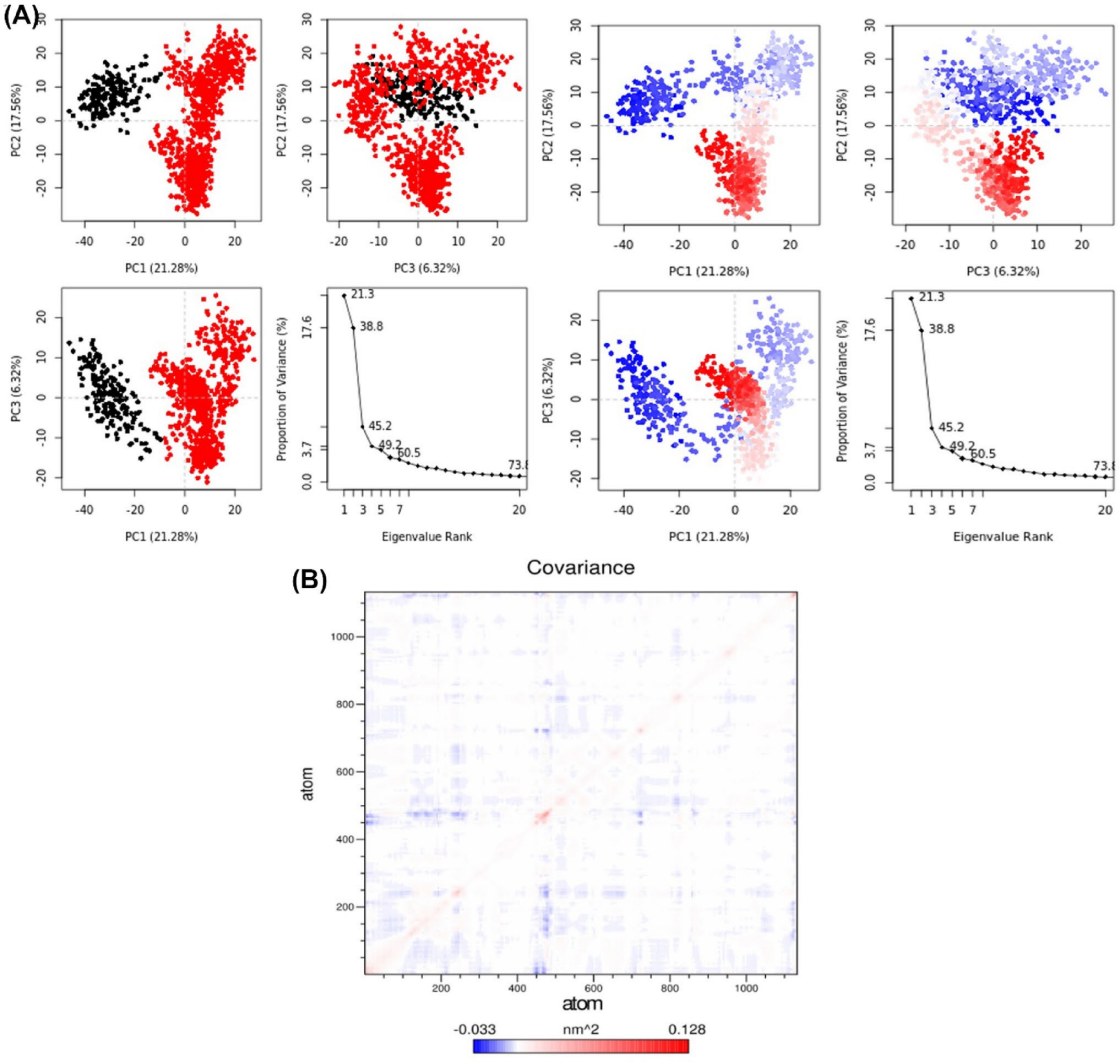
**A** The principal component analysis, indicating fluctuations in different hyperspaces, and **B** PCA and Covariance analysis of lead compound-PTP1B complex, showing the major conformational motions and correlated atomic furcation, ’s in is also mentioned in MD simulations

Projection of MD trajectories of Principal Component Analysis (PCA) revealed distinct conformational distributions for the three systems. The apo PTP1B protein occupied a relatively broader conformational space, indicating the enhanced flexibility and diverse dynamic behavior in the absence of a ligand. In contrast, the lead compound-bound systems exhibited more confined and well-defined clusters along PC1-PC2 and PC1-PC3 projections, suggesting reduced conformational freedom upon ligand binding (Fig. 16). To explore correlated and anti-correlated motions between residues, a dynamic cross-correlation (covariance) matrix was generated from the MD trajectories. The apo protein displayed a mixture of correlated (positive) and anti-correlated (negative) motions across distant residue pairs, indicative of higher internal flexibility.

#### MM/GBSA

**Fig. 17:**
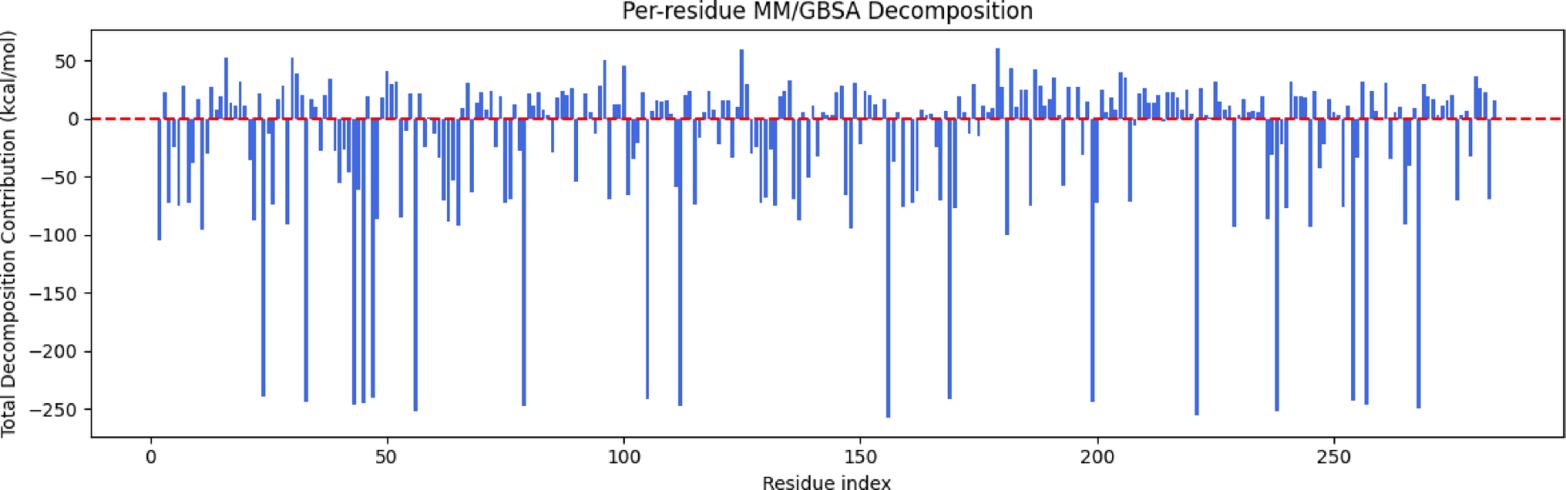
Per-residue MM/GBSA binding decomposition highlighting key energetic residues with strong favorable contributions to the ligand binding, indicating a stable interaction

The plot shows residue-wise MM/GBSA contributions ranging approximately from + 40 to − 250 kcal mol^−1^, with the majority of residues clustered near − 10 to + 20 kcal mol^−1^ and a limited number of residues exhibiting very strong favorable contributions (< − 100 kcal mol^−1^). These deep negative minima represent energetic hotspots, indicating that binding free energy is dominated by a small subset of residues rather than being uniformly distributed across the protein surface. This energetic pattern is characteristic of drug-like binding because small-molecule drugs typically achieve high affinity through specific, localized interactions (hydrophobic packing, π-π stacking, and key electrostatic contacts) within a defined pocket, rather than through widespread weak interactions (Fig. 17). The presence of a few but intense stabilizing residues, combined with many near-neutral contributions, implies high binding specificity, optimal pocket complementarity, and favorable enthalpy-entropy balance, all of which are core features of drug-like ligands suitable for further optimization.

The MM/GBSA results revealed a total binding free energy (ΔG_bind_) of − 13.78 ± 0.18 kcal mol^−1^, showing a favorable and stable interaction between the ligand and protein. The binding is mainly dominated by van der Waals forces (− 35.25 kcal mol^−1^), with additional stabilization from electrostatic interactions (− 5.21 kcal mol^−1^) (Table 4). Although the polar solvation energy (+ 31.15 kcal mol^−1^) unfavorably contributes to the system, it is outweighed by strong gas-phase interactions (ΔGGAS = − 40.46 kcal mol^−1^) and favorable non-polar solvation (− 4.46 kcal mol^−1^). Overall, these findings suggest that hydrophobic interactions play a key role in stabilizing the protein–ligand complex.

**Table 4:** Energy decomposition of MM/GBSA binding free energy for the lead compound-PTP1B complex

Energy Component	ΔG (kcal/mol)	SEM (kcal/mol)
ΔVDWAALS	35.25	0.22
ΔE_EL_	− 5.21	0.30
ΔG_GAS_	− 40.46	0.39
ΔE_GB_	31.15	0.34
ΔE_SURF_	− 4.46	0.03
ΔG_SOLV_	26.68	0.32
ΔG_bind_ (ΔTotal)	− 13.78	0.18

#### Hydrogen bond occupancy analysis

To further evaluate the persistence and stability of intermolecular interactions within the protein-lead complex, hydrogen bond occupancy analysis was performed over a 200 ns MD simulation. This represents the proportion of simulation time during which a specific hydrogen bond is maintained and serves as an indicator of the stability of the interaction. A hydrogen bond between the lead compound (LIG284-Side) and the side chain of ASP240 was observed with an occupancy of 0.79%, indicating the interaction is highly transient and occurs only sporadically throughout the simulation. Typically, high occupancy hydrogen bonds are associated with stable and functionally significant interactions, whereas low occupancy bonds represent weak and short-lived contacts resulting from dynamic structural fluctuations. Accordingly, the ASP240 interaction does not appear to play a major stabilizing role within the binding pocket. Overall, results suggest that the stability of the protein-lead ligand complex is not primarily driven by persistent hydrogen bonding, but rather by other interactions such as van der Waals and hydrophobic interactions, consistent with MM/GBSA energy decomposition findings.

#### Aromatic interaction analysis

**Fig. 18:**
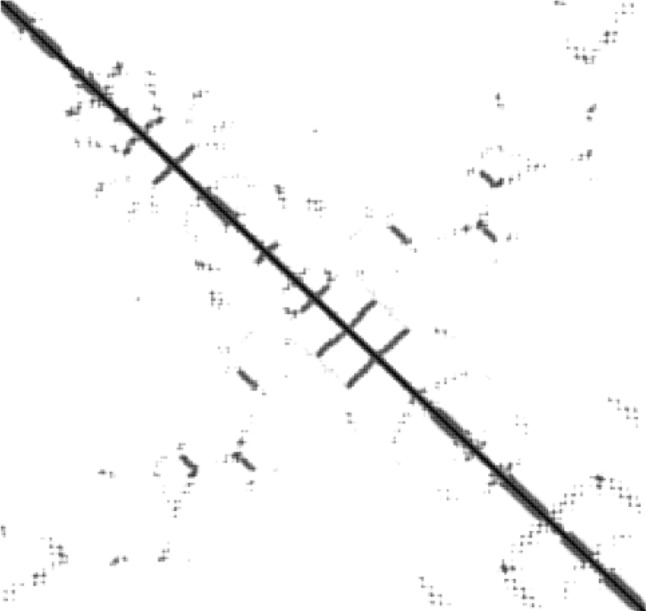
The aromatic contact map of the lead compound-PTP1B complex

Aromatic contact map analysis was performed to investigate the contribution of aromatic interactions to the stability of the protein-ligand complex during the molecular dynamics simulation. Aromatic interactions, including π–π stacking and related non-covalent contacts, play an important role in stabilizing ligand binding by providing hydrophobic and electronic complementarity within the binding environment. In the aromatic contact map (Fig. 18), the diagonal line represents the sequential arrangement of residues within the protein structure, where each position corresponds to a residue index.

The short projections appearing along the diagonal indicate time points at which aromatic contacts were formed between the ligand and residues of the protein throughout the simulation trajectory. The distribution of these marks across different regions of the diagonal suggests that several aromatic interactions occurred during the simulation. Areas with relatively higher densities of marks indicate contacts that appeared more frequently and persisted for longer durations, whereas sparsely distributed marks represent transient interactions arising from conformational fluctuations. Overall, the analysis demonstrates that multiple aromatic contacts were formed during the simulation, supporting the stabilization of the protein-ligand complex and contributing to the overall binding persistence observed across the trajectory.

### Drug’s toxicity

**Fig. 19:**
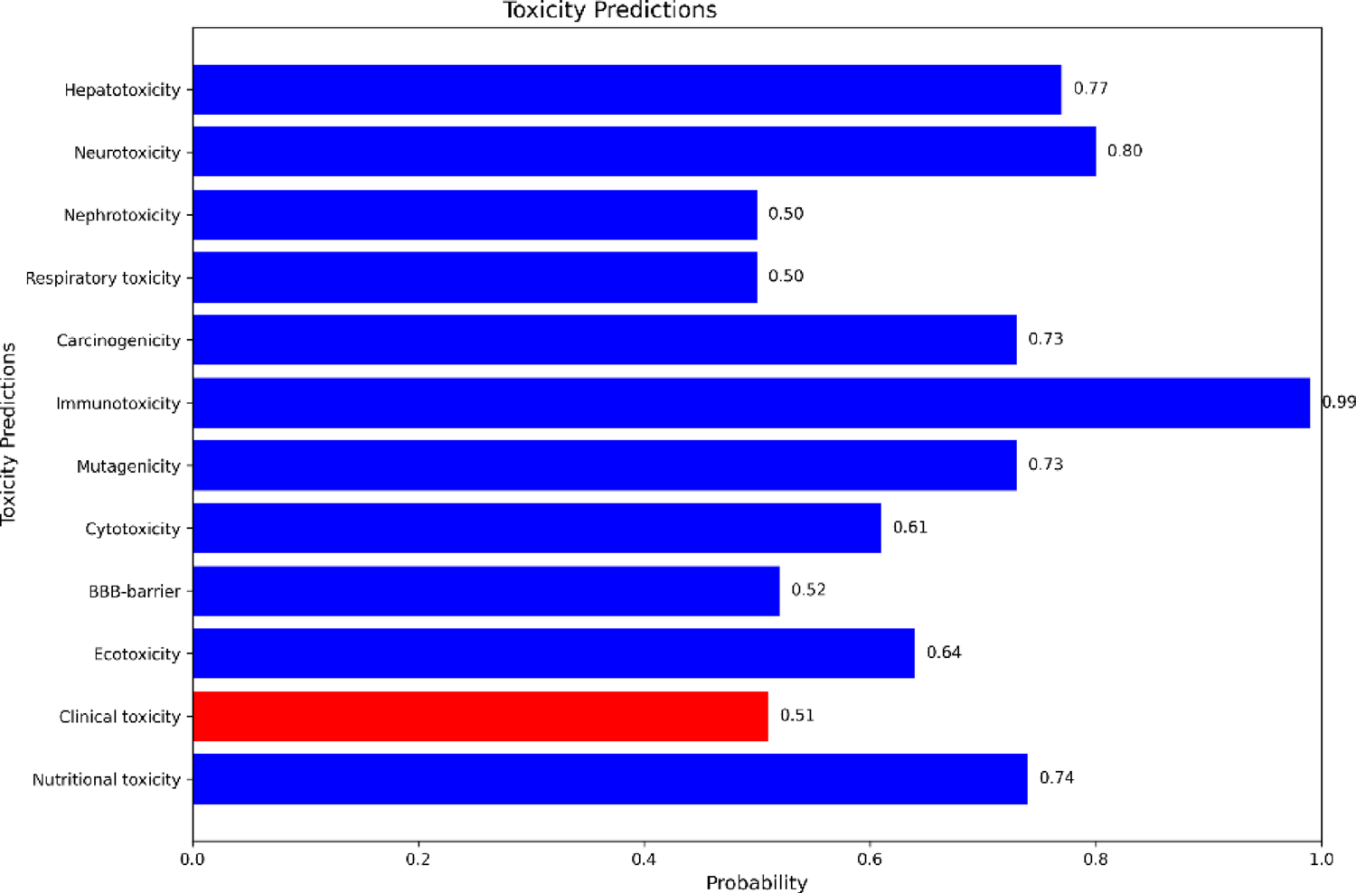
Bar graphs displaying the prediction of the toxicity of the lead compound. Active predictions were plotted as red bars, and Inactive predictions were plotted as blue bars, with the corresponding probability displayed on the side

To confirm the safety and feasibility of these compounds for further development, an in silico toxicity evaluation was performed using the ProTox-3.0 webserver. The outcome showed that the compounds had a good toxicity profile, with negative results for hepatotoxicity, neurotoxicity, nephrotoxicity, respiratory toxicity, carcinogenicity, immunotoxicity, mutagenicity, and cytotoxicity, as well as a cutoff score of 0.5–0.99 (inactive), as they were confirmed to be “Inactive” (Fig. 19). This implies that liver-specific or systemic toxicity is unlikely for the compounds. The side effect “clinical toxicity” (probability of occurrence 0.51). This is the only clinical toxicity predicted as “Active”, but its low probability suggests it is a marginal prediction that may not have a significant impact on the overall safety profile. Combined with the lack of carcinogenic, mutagenic, and organ-specific toxicity, indicates that the compounds described are within acceptable safety limits and therefore may be less likely to suffer from adverse effects in preclinical or clinical studies (Table 5). The structure-activity relationships will vary throughout, resulting from local (tissue) availability occurring during drug metabolism at these three metabolic positions. These results apparently indicate that the compounds can serve as promising, safe therapeutic agents for further pharmacological studies.

**Table 5:** Expected activity scores for the lead compound, toxicity prediction

Sl. No.	Target	Prediction	Probability
1	Hepatotoxicity	Inactive	0.77
2	Neurotoxicity	Inactive	0.80
3	Nephrotoxicity	Inactive	0.50
4	Respiratory toxicity	Inactive	0.50
5	Carcinogenicity	Inactive	0.73
6	Immunotoxicity	Inactive	0.99
7	Mutagenicity	Inactive	0.73
8	Cytotoxicity	Inactive	0.61
9	BBB-barrier	Inactive	0.52
10	Ecotoxicity	Inactive	0.64
11	Clinical toxicity	Active	0.51
12	Nutritional toxicity	Inactive	0.74

Active/Inactive and corresponding probability

### VEGA QSAR, and ADMET analysis

The inhibitors were further analyzed using VEGA QSAR to predict both pharmacokinetic and toxicological profiles, including skin irritation potential, P-gp activity, mutagenicity (Ames test), plasma protein binding, and total body elimination half-life (Table 6). The model also predicted no skin irritation, no mutagenicity in the Ames test, and no inhibition of P-glycoprotein. Plasma protein binding was relatively low (logK = 0.0164), and the elimination half-life was also consistent, having a value of 1.158 h. None of the compounds had any toxicological alerts, so they may be considered safe to biological systems. In general, the VEGA QSAR results are in line with the good ADMET profile of this series, such as interesting risk factors (no skin irritation, mutagenicity, or P-glycoprotein effect), and equal stability/reactivity without impairment on drug-likeness (without excess of Lipinski, suggesting potential BBB penetration). These findings highlight the clinical potential of the scaffold and direct further optimization of PTP1B inhibitor design.

**Table 6:** Pharmacokinetic and toxicological profiles of the lead compound from VEGA QSAR

Compound	1
Skin irritation	No
Mutagenicity (Ames test)	No
P-glycoprotein activity	Inactive
Plasma protein binding [logK]	0.0164
Total body elimination half-life (Hour)	1.158

**Fig. 20:**
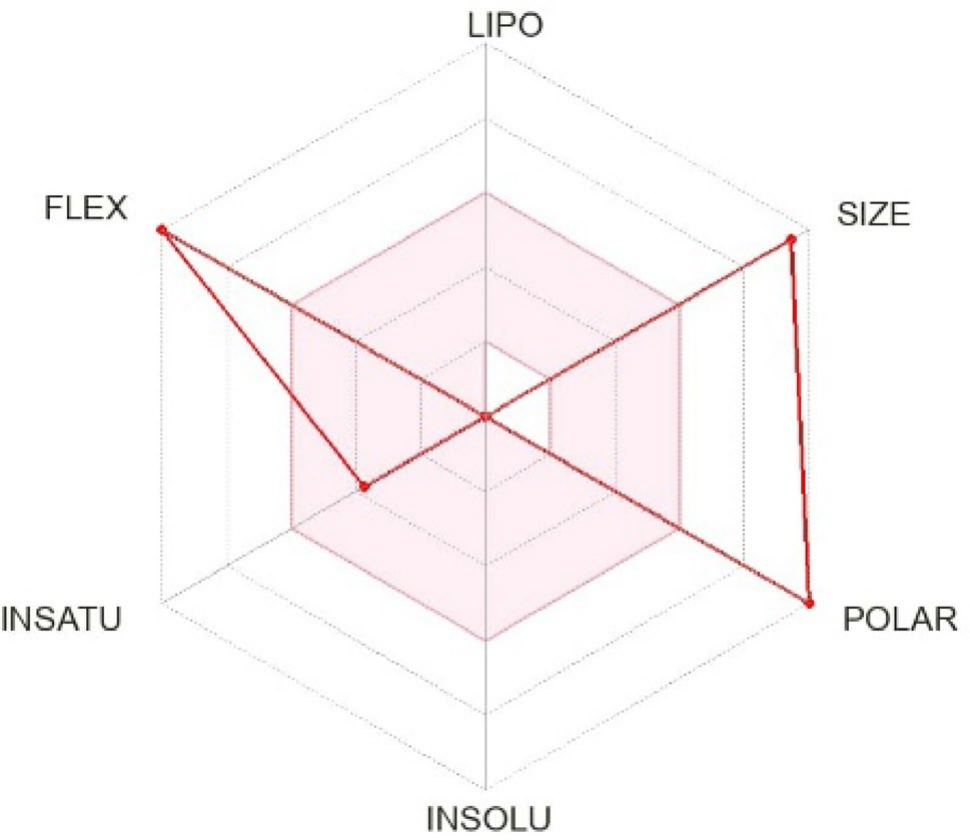
SwissADME bioavailability radar of lead compound. The red scale shows the compound’s physicochemical properties across six key dimensions (lipophilicity, size, hydrogen bonding, solubility, aromaticity, and flexibility). The pink zone represents the optimal drug-like space

ADMET properties of the top hit were evaluated using the SwissADME online tool. Drug-likeness assessment was carried out by analyzing parameters such as Lipinski’s rule violations, water solubility, lipophilicity (LogP), blood-brain barrier penetration, gastrointestinal absorption, and cytochrome P450 inhibition profile (Table 7). The bioavailability radar provided a quick visualization of six key physicochemical features: lipophilicity, size, polarity, solubility, saturation, and flexibility required for drug-likeness (Fig. 20). The red line represents the compound’s physicochemical space, while the pink region indicates the optimal drug-like range. A plot outside the pink region indicates lower drug-likeness.

**Table 7:** ADMET profiling result of lead compound, determined by SwissADME, showing its physicochemical and pharmacokinetic parameters relevant to drug-likeness and oral bioavailability

Property	Value
Molecular formula	C_32_H_47_N_7_O_15_S
Num. H-bond acceptors	16
Num. H-bond donors	11
LogP (consensus)	-2.46
Water solubility (ESOL)	0.53 (Highly soluble)
GI absorption	Low
BBB permeant	No
P-gp substrate	Yes
Lipinski violations	3 violations
Bioavailability score	0.11
PAINS alerts	0 alert
Synthetic accessibility	6.42
Leadlikeness	No; 2 violations: MW > 350, Rotors > 7

## Discussion

A combination of computational techniques served as the basis for the current research, which aims to identify the most promising PTP1B inhibitors. The enzyme in question plays a central role in the signaling pathways of leptin and mosquitoes and is thought to be a major driver of T2DM onset. By applying a range of computational techniques, including virtual screening, molecular docking, analogue refinement, ADMET prediction, toxicity assessment, molecular dynamics (MD) simulation, and DFT analysis, the researchers proposed a robust method for assessing the therapeutic potential of the newly identified scaffolds. The virtual screening of 1000 antidiabetic compounds provided a rapid route to the most probable candidates with good affinity for the active site of PTP1B. The PubChem CID 44560696 was initially considered the best hit, but after a deeper study of its structure, CID 44560744 was found to be even more effective, with an affinity of − 9.13 kcal/mol. The greater binding energy of CID 44560744 indicates how even a slight modification in their structure could significantly influence the recognition by the protein of the ligand, particularly within its highly conserved active site, i.e., the PTP1B pocket. After structure-based virtual screening, the lead compound was subjected to DFT calculations to obtain its fully optimized geometry, which was subsequently re-docked into PTP1B active site to refine the binding analysis. The DFT and FMO calculations were also complementary in assessing the compound’s electronic behavior. The very high HOMO-LUMO band gap (7.168 eV), which indicated high stability and represents resistance of the molecule toward charge transfer. The low softness and moderate hardness of the compound, as calculated computationally, are also indicators that the compound can form stable electrostatic interactions with the binding site. Meanwhile, the compound should be an electron acceptor (due to its slight positive electrophilic index) and is intermediate via enzyme inhibition, which can be reversed. The DFT optimized structure was subsequently subject to molecular docking to analyze its interactions with key amino acid residues. The ligand was also observed to form several hydrogen bonds with key catalytic residues (ARG24, ARG221, GLN262 and ASP48), which are crucial for phosphatase inhibition. In addition, hydrophobic interactions alkyl, π -alkyl, π -sulfur and π -sigma involved in the stability of the ligand but also indicated a good fit between the corresponding compounds and the binding site of the enzyme.

The two-stage docking was unsuccessful; however, re-docking of the native ligand yielded a tight agreement with the crystal structure, supporting the correctness of our docking protocol. This was followed by an interaction profile of CID 44560744, which showed that it had the potential to be selective through binding to the subpockets (B, C, and D) surrounding the active site- this is an essential criterion for selective PTP1B inhibition. The molecular docking was also performed with references inhibitor ursolic acid. The results showed ursolic acid was well situated in the catalytic pocket of PTP1B, exhibiting a calculated binding affinity of − 6.34 kcal mol^−1^. Notably binding affinity was lower than that of lead compound, indicating that the screened compound possess superior binding potential toward PTP1B. To gain insight into dynamic stability in addition to the static docking results, MD simulations were performed for both the apo protein and the ligand-protein complex. The PTP1B-CID 44560744 complex established stable RMSD profiles following initial equilibration, and no significant conformational changes were observed throughout the 200 ns of simulation that could be attributed to binding interactions. An attractive indication of enzyme dynamics is the absence of evident loop fluctuations upon binding, as seen in RMSF plots. The Rg and SASA values also suggested that the complex was more compact than the non-ligand bound form, which represented the stronger structure after ligand binding. Moreover, the hydrogen-bond arrangement throughout the simulation also confirmed the compound’s good kinetics (or steady binding). The MD results indicate that lead compound is both energetically and structurally stable under physiological conditions. Persistent hydrogen bonding with the solvent throughout simulation reflects good aqueous solubility and physicochemical robustness, while stabilized RMSD profile confirms conformational integrity after initial relaxation. The PCA and covariance analyses show that lead compound binding restricts conformational freedom of PTP1B compared to apo state, suggesting protein stabilization upon the formation of complex. Additionally, MM/GBSA residue decomposition reveals that binding is dominated by a minimum number of energetic hotspots, a hallmark of drug-like specificity and high-affinity interactions. The drug-like characteristics and safety pharmacology findings indicated that the compound had a favorable toxicological profile. ProTox-II and VEGA-QSAR also ruled out the hazards of hepatotoxicity, mutagenicity, carcinogenicity, immunotoxicity, and neurotoxicity, stating that there was no high risk. Although SwissADME predicted low gastrointestinal absorption and identified multiple Lipinski’s rule violations (most likely due to the compound’s high size and polarity), these liabilities are not distinctive for peptide-like inhibitors targeting PTP1B, whose catalytic site is highly polar. Whether the pharmacokinetic limitations observed in these latter studies can be circumvented by either structural optimization or formulation strategies was unclear. The calculated results provide strong evidence that CID 44560744 is an excellent lead scaffold for PTP1B inhibition. All of these combined illustrate the great potential for further improvement, with this compound’s high binding affinity, reversible dynamic stability, relative nontoxicity, and strong electronic reactivity. Further structural optimization to improve drug-like properties, including in vitro enzymatic tests for inhibitory activity and, perhaps, in vivo testing for therapeutic efficacy, is warranted.

## Conclusion

This investigation employed a systematic, efficient in silico research workflow to comprehensively screen, optimize, and elucidate the mechanisms of protein tyrosine phosphatase 1B (PTP1B) inhibitors, ultimately identifying the promising candidate compound CID 44560744. Starting with a large-scale virtual screening, this work conducted a structural basis screening of 1000 anti-diabetic-related molecules from PubChem and ChEMBL, identifying the initial high-affinity molecule, CID 44560696. Subsequently, using CID 44560744 as a template, a structural analogue search was conducted, followed by further molecular docking screening. CID 44560744 exhibited a superior binding energy (-9.13 kcal/mol) and a stable binding conformation. DFT optimized structure was used for molecular redocking of lead compound. It formed multiple hydrogen bonds and hydrophobic interactions with key catalytic residues (e.g., Asp181, Arg221, and Gln262 etc.,), demonstrating strong active-site affinity and structural adaptability. Comparative docking with reference inhibitor ursolic acid showed a lower binding affinity (− 6.34 kcal mol^−1^) than the lead compound, confirming the superior binding potential of screened compound toward PTP1B. Docking backtesting experiments showed a high degree of overlap between the predicted conformation and the crystal structure, demonstrating the reliability of the current docking process. To further confirm its binding stability, subsequent 200 ns molecular dynamics simulations showed that the PTP1B-CID 44560744 complex exhibited a stable trend across RMSD, RMSF, Rg, and SASA indices. The complex structure was more compact, demonstrating its binding tolerance in dynamic environments, which is beneficial for its inhibitory effect. Hydrogen bond timing analysis also showed that the molecule can maintain key interactions over a long period, fully supporting its stable binding ability. At the electronic structure level, calculations based on DFT and FMO further revealed the molecular electronic behavior. The results showed that the compound has an extremely high HOMO-LUMO band gap (7.168 eV), indicating high electron mobility and stability, which is conducive to forming stable electrostatic and polar interactions with the enzyme catalytic pocket. Simultaneously, its low softness and moderate charge-transfer ability reflect its good electronic regulation properties and potential stable inhibitory effect. In terms of pharmacokinetics and toxicology, combined evaluations using SwissADME, ProTox-II, and VEGA-QSAR showed that CID 44560744 exhibits good water solubility, is non-carcinogenic, and has no significant hepatotoxicity or genotoxicity, demonstrating a high overall safety profile. Although it deviates somewhat from Lipinski’s rules and has low gastrointestinal absorption, this highly polar, large-molecular-weight structure is standard among PTP1B inhibitors. It can be further improved through subsequent structural optimization, modification, or drug-delivery strategies.

In summary, this study successfully identified CID 44560744 as a potential high-efficiency PTP1B inhibitor, demonstrating advantages in binding affinity, kinetic stability, electronic structural characteristics, and toxicological safety. These results lay a solid foundation for its potential as a lead compound for the treatment of type 2 diabetes. This study also demonstrates the importance of in silico multi-technology fusion strategies in the discovery of novel PTP1B inhibitors. In the future, we should combine in vitro enzyme assays, cell models, and in vivo efficacy evaluations to further verify its inhibitory activity, specificity, and pharmacokinetic properties, and to carry out necessary structural modifications to advance it toward clinical candidate status.

## Data Availability

All data generated or analyzed during this study are included in this published article. Further datasets or simulation files can be obtained from the corresponding author upon reasonable request.
